# Normal stages of embryonic development of a brood parasite, the rosy bitterling *Rhodeus ocellatus* (Teleostei: Cypriniformes)

**DOI:** 10.1002/jmor.21335

**Published:** 2021-04-02

**Authors:** Wenjing Yi, Martin Rücklin, Robert E. Poelmann, David C. Aldridge, Michael K. Richardson

**Affiliations:** ^1^ Institute of Biology University of Leiden, Sylvius Laboratory Leiden the Netherlands; ^2^ The Key Laboratory of Aquatic Biodiversity and Conservation, Institute of Hydrobiology Chinese Academy of Sciences Hubei China; ^3^ Vertebrate Evolution, Development and Ecology Naturalis Biodiversity Center Leiden The Netherlands; ^4^ Department of Zoology University of Cambridge Cambridge UK

**Keywords:** co‐evolution, embryogenesis, hatching, morphogenesis, staging

## Abstract

Bitterlings, a group of freshwater teleosts, provide a fascinating example among vertebrates of the evolution of brood parasitism. Their eggs are laid inside the gill chamber of their freshwater mussel hosts where they develop as brood parasites. Studies of the embryonic development of bitterlings are crucial in deciphering the evolution of their distinct early life‐history. Here, we have studied 255 embryos and larvae of the rosy bitterling (*Rhodeus ocellatus*) using in vitro fertilization and X‐ray microtomography (microCT). We describe 11 pre‐hatching and 13 post‐hatching developmental stages spanning the first 14 days of development, from fertilization to the free‐swimming stage. In contrast to previous developmental studies of various bitterling species, the staging system we describe is character‐based and therefore more compatible with the widely‐used stages described for zebrafish. Our bitterling data provide new insights into to the polarity of the chorion, and into notochord vacuolization and yolk sac extension in relation to body straightening. This study represents the first application of microCT scanning to bitterling development and provides one of the most detailed systematic descriptions of development in any teleost. Our staging series will be an important tool for heterochrony analysis and other comparative studies of teleost development, and may provide insight into the co‐evolution of brood parasitism.

## Graphical abstract

A selection of microCT images of rosy bitterling embryos.

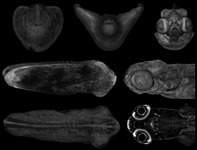

## 1 INTRODUCTION

The bitterlings are a group of freshwater teleost fish in the family Acheilognathidae (Teleostei: Cypriniformes) which have a brood parasitic life‐history (Smith et al., 2004). With their elongated ovipositor, female bitterlings lay eggs in their preferred mussel host species through the exhalant siphon of the mussel (Boeseman et al., 1938; Duyvené de Wit, 1955; Olt, 1893; Rouchet et al., 2017; Wiepkema, 1962). The eggs are introduced directly into the gill chamber by the ovipositor (Chang, 1948). They come to occupy the lumen of the water tube between the gill lamellae (Kim et al., 2008; Kim et al., 2017; Tankersley, 1996). Later, male bitterlings release their sperm near the inhalant siphon (Boeseman et al., 1938; Smith & Reichard, 2013). The sperm are carried into the mussel by the water flow and fertilize the eggs; subsequently the eggs undergo their early stages of development inside the gill chamber (Liu et al., 2006; Reichard et al., 2007). When the larvae are capable of swimming, they migrate into the exhalant cavity and emerge from the host; this marks the end of the parasitic phase of their life (Aldridge, 1999).

Because of their interesting life history, bitterlings have been intensively studied by ecologists and behavioral biologists (Boeseman et al., 1938; Smith et al., 2004; Wiepkema, 1962). Aspects of the bitterling life cycle that have been studied include the parental behavior of bitterlings (Reichard et al., 2004) and bitterling‐mussel co‐evolution (Mills & Reynolds, 2003). For studies of bitterling and mussel phylogeny, see Chang et al. (2014) and Kawamura et al. (2014). While there have been many studies of adult bitterlings, their development is less well‐known. This is largely because of the difficulties of accessing the early embryos inside the mussel host. Furthermore, when early embryos are dissected out of the mussel, it is difficult to stage them because the precise time of fertilization is unknown (Duyvené de Wit, 1955; Olt, 1893). The unique early life history of bitterlings means that the embryos are subject to a protected but physiologically challenging environment, which may result in deviations from typical teleost embryonic development (Aldridge, 1999). The first stage description of the early life history of the European bitterling, *Rhodeus amarus*, was given by Olt (1893). Olt (1893) noticed the peculiar forms of the yolk and presented the changing shapes of the yolk stage by stage in illustrations, whereas he failed to give precise developmental age of these stage. Fortunately, the techniques of in vitro fertilization and time‐controlled in vitro incubation were used to obtain embryos of the most widely‐distributed oriental bitterling, the Rosy bitterling, *Rhodeus ocellatus* (Chang, 1948; Chang & Wu, 1947;Kim & Park, 1985; Nagata & Miyabe, 1978; Park & Han, 2018). Previous studies provided detailed descriptions (Chang, 1948; Chang & Wu, 1947), as well as schematic developmental atlases of bitterling embryonic stages based on external morphological characters (Kim & Park, 1985; Nagata & Miyabe, 1978; Park & Han, 2018).

Nagata and Miyabe (1978) described 30 developmental stages of *R*. *ocellatus*: 14 stages before hatching; and 16 post‐hatching, "prelarvae” stages. Unfortunately, the illustrations provided by Nagata and Miyabe (1978) are not annotated. Kim and Park (1985) described 20 pre‐hatching stages with special emphasis on the period from yolk plug closure to presence of the tailbud (stages O–S in their series). They successfully illustrated the migration and elongation of the rostral end towards the ventral side but did not indicate the rostral‐caudal axis in their illustrations, which leaves some descriptions ambiguous.

The developmental age was recorded by Nagata and Miyabe (1978) in terms of hours post fertilization (hpf). Kim and Park (1985) and Park and Han (2018) also used the term hpf for pre‐hatching stages, but used "hours post hatching” (hph) and "days post hatching” (dph) for later stages. Those three studies differed in the timing that they assigned to certain developmental events. For example, "hatching” was at 27.8 hpf, 39 hpf, and 50 hpf in Nagata and Miyabe (1978), Kim and Park (1985) and Park and Han (2018), respectively. We suggest that this difference in the time of hatching recorded in these first two studies is because the rearing temperature was different, namely: 22 ± 1°C in Nagata and Miyabe (1978), 17–25.5°C in Kim and Park (1985). It is well‐known that temperature influences developmental timing (Werneburg, 2009). The greatest difference in time of hatching is between Park and Han (2018) and Nagata and Miyabe (1978), although the rearing temperature in Park and Han (2018) is 21.5 ± 1°C, more similar to Nagata and Miyabe (1978). It is because the newly‐hatched embryo illustrated by Park and Han (2018) corresponds to a later developmental stage in Nagata and Miyabe (1978). Because Kim and Park (1985) used a relatively wide temperature range, we have chosen to use here the developmental age in Nagata and Miyabe (1978) as a guide. In Nagata and Miyabe (1978) and Kim and Park (1985), hatching occurred at the same morphological stage, namely when the embryo has 6–10 somites, Kupffer's vesicle is present, and the tailbud is not yet free from the yolk extension.

In the literature on teleost development, it is sometimes stated that the embryo becomes a larva at hatching (e.g., Ali et al., 2011; Ballard, 1981). Kunz (2004) noticed that bitterlings have an ostraphilic reproductive habit (laying eggs in mussels) and have a nidicolous (nest‐dwelling) type of hatching. This means that they hatch at a relatively early age and are not capable of independent living by means of, for example, free‐swimming and foraging (Aldridge, 1999; Li & Arai, 2010). Therefore, the term "postembryo" is suggested by Kunz (2004) to describe the fish after hatching until the yolk is completely absorbed. After that, when exogenous feeding begins, the fish is termed a larva.

**FIGURE 1:**
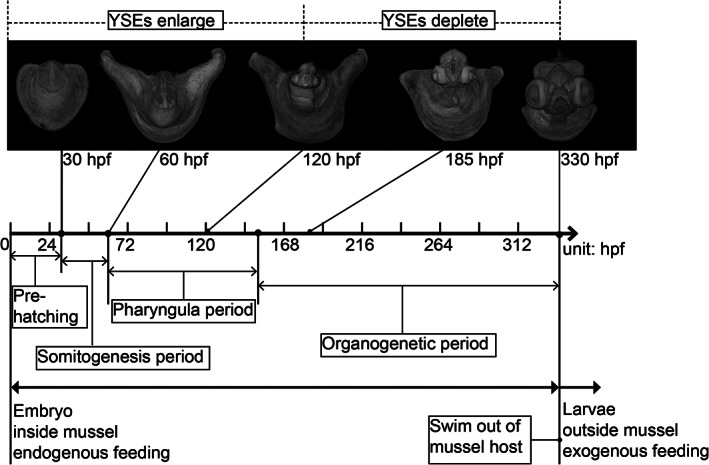
*Rhodeus ocellatus*, the development timeline. Abbreviations: hpf, hours post fertilization; YSEs, yolk sac extensions

Here, we shall define the embryonic period of bitterlings as beginning at fertilization and ending when the embryos are capable of swimming out of their mussel host. Post‐hatching individuals are termed "embryos" instead of "larvae" in this study (Figure 1). Hatching, in this view, implies the breaking of the chorion, and the embryo‐to‐larva transition is a nutritional definition (when endogenous feeding transitions to exogenous feeding).

In the current study, we make detailed descriptions of developmental stages in *R*. *ocellatus* in order to extend the published studies. For post‐hatching stages, we use microCT (X‐ray microtomography) to reveal the internal structure of the embryos in tomographic sectional view, and to provide 3‐D (three‐dimensional) visualization of the external morphology of development. In preliminary studies (data not shown) we found that the perivitelline space of bitterlings is quite narrow which means that there is little room to perform dechorionation with forceps without damaging the yolk or embryo. Therefore, we decided not to dechorionate the eggs. Because the chorion is highly impermeable to many reagents (Masuda et al., 1986; Masuda et al., 1992), we assumed that the contrast medium needed for microCT would not penetrate. Therefore, we decided not to use microCT for the pre‐hatching stages.

Advantages of the microCT technique are that it is less time‐consuming than conventional histological sectioning, it does not destroy the samples, and larger specimens can be studied than is possible with, for example, confocal microscopy (Bassi et al., 2015; Huisken & Stainier, 2009; Weber et al., 2014). The main disadvantages of microCT are a lack of cellular resolution, the inability to use special stains to identify particular structures or molecules, and the inability to perform in vivo tracing. Metscher (2009) has pioneered the application of microCT to developmental biology by developing a soft‐tissue staining protocol. MicroCT has been used to study mouse development (see for example the 3‐D mouse embryo atlas (Wong et al., 2012), compare phenotypic variation of larval and juvenile zebrafish at the histological level (Ding et al., 2019), and quantitative morphometric analysis of adult teleost fish (Weinhardt et al., 2018).

By analyzing a developmental series of the rosy bitterling, with 3‐D and sectional views, we hope to provide a practical guide to staging bitterlings embryos in the lab and field, and provide a knowledge foundation for future research that focusses on development, comparative embryology, evolutionary developmental biology (evo‐devo), and gene expression patterns. Our study may also serve as a model in the emerging discipline of eco‐evo‐devo, or ecological evolutionary developmental biology, which aims to integrate evolution and development with ecology (Abouheif et al., 2014; Gilbert et al., 2015).

## 2 MATERIALS AND METHODS

### 2.1 Animal husbandry

Rosy bitterlings (*Rhodeus ocellatus*, Kner, 1866) were kept in indoor freshwater aquaria with controlled light and temperature. Adult fish were purchased from Ruinemans Aquarium B.V., Montfoort, NL. At 06:45 a.m. and 08:15 p.m., the lights were turned on and off, respectively, with a 15 min transition time to avoid sudden shock. The room temperature and water temperature were kept at 22.5 ± 1°C and the fish were checked every day by caretakers. Fish were fed with frozen chironomid larvae (Ruinemans Aquarium B.V., Montfoort, NL) daily. Duck mussels (*Anodonta anatine*, Linnaeus, 1758) and swan mussels (*Anodonta cygnea*, Linnaeus, 1758) were obtained from Vijver‐centrum Enschede, Aquaria Veldhuis; Enschede, NL, and kept indoors with natural light from a window, in a shallow filtration tank without feeding. Water from the filtration tank was led into the fish tanks to stimulate the bitterling mating behavior.

### 2.2 In vitro fertilization

Embryos with synchronized development were obtained by in vitro fertilization following the method of Nagata and Miyabe (1978). Briefly, sexually mature parental fish were chosen based on the bright mating color of the male and the elongated ovipositor of the female. Eggs were expressed from 35 females into a clean, dry 10 cm Petri dish by gentle abdominal compression. Sperm was also harvested from 25 males by gentle abdominal compression. We used a narrow‐mouthed pipette to distribute the sperm evenly over each batch of eggs in a clean Petri dish. Fresh aquarium water was then added, so as to synchronously activate embryonic development. Embryos were raised 20 per Petri dish containing embryo water (Kimmel et al., 1995) changed every 24 h. The Petri dish was kept in an incubator with stationary shelves at 22.5 ± 1°C.

### 2.3 Time‐lapse videography

For all pre‐hatching stages, we used time‐lapse videography of embryos at room temperature with epi‐illumination from a fiber‐optic lamp (Schott KL 1500 LCD). Photos were taken every 5 min with a CCD (charge‐coupled device) camera (Nikon DS‐Fi1‐L2) connected to stereo microscope (Nikon SMZ1500). The images acquired had a minimum resolution of 300 dpi (dots per inch) and were stored in JPG format. During the recording, embryos were kept in glass embryo dishes (uncovered, 30 mm diameter × 12 mm deep) filled with embryo water. Because young embryos do not yet show spontaneous movements, it was not necessary to immobilize them with agarose embedding or anesthesia. The water level during the recording period was maintained by adding drops of egg water to the embryo dishes as necessary.

### 2.4 Embryo fixing, processing, and microCT scanning

**FIGURE 2:**
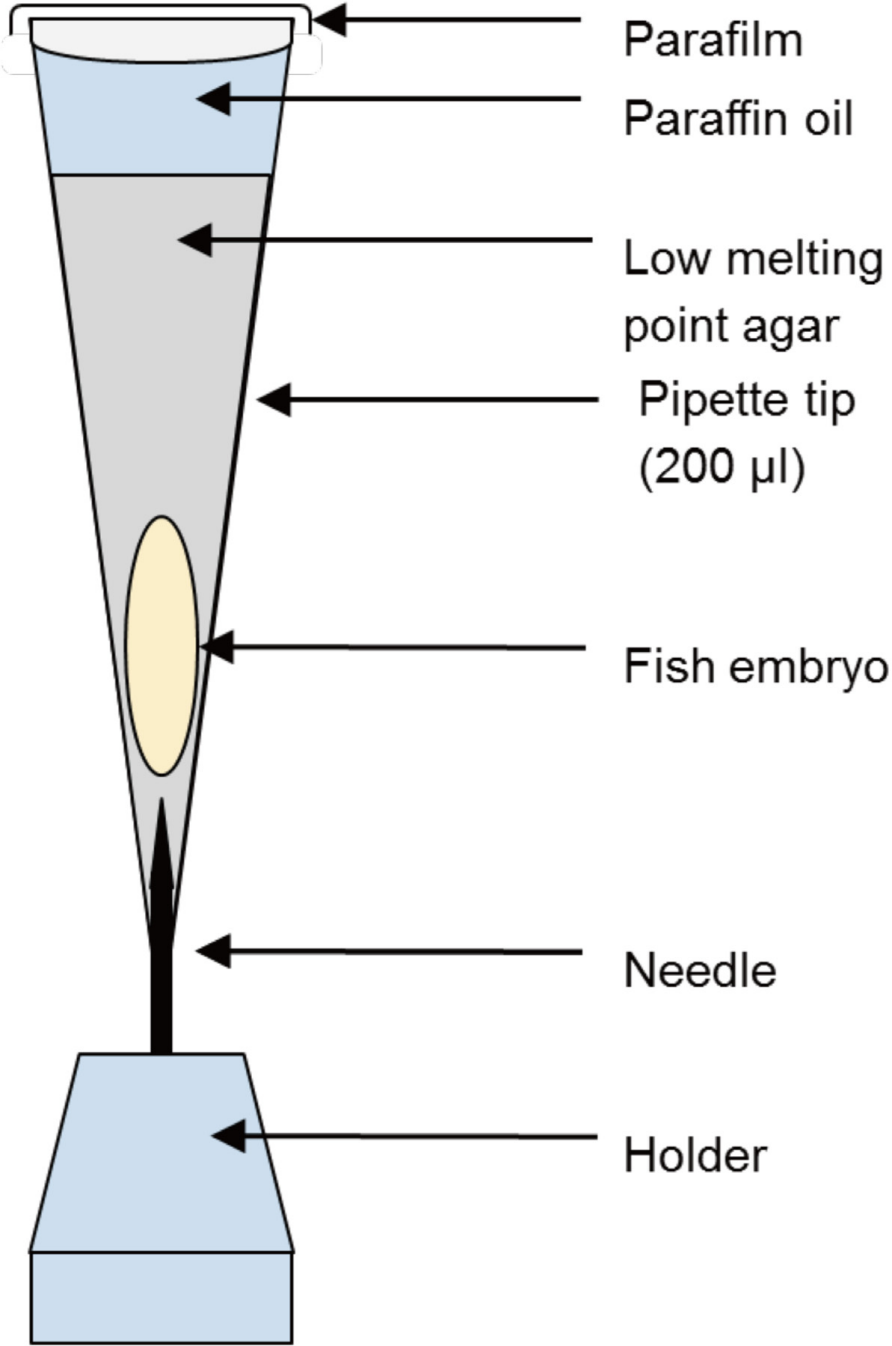
Schematic illustration of the embedding setup for microCT

The following protocol is based on Metscher (2009), and Babaei et al. (2016). Post‐hatching stages were fixed for microCT at different developmental time points as shown in Table 1. The fixative was 3% paraformaldehyde (pFA) and 1% glutaraldehyde (GA) in 0.1 mol L^−1^ phosphate buffered saline (PBS), pH 7.0 at 4°C overnight. After rinsing in PBS (2 × 10 min), specimens were stained with iodine‐potassium iodide (1% iodine in 2% potassium iodide) for 12 h or phosphotungstic acid (PTA, 0.3% phosphotungstic acid in 70% ethanol) for ≥24 h. Staining was carried out on a rotary mixer at 6 revolutions per minute. After staining, the embryos were stored at 4°C in 70% ethanol. Samples were immobilized in 1% low melting‐point agarose, sealed with paraffin oil and parafilm, and stabilized in a polystyrene tube during scanning (see Figure 2).

**TABLE 1:** Micro‐CT scanning parameters for embryos during post‐hatching stages

Age (hpf)	Stage name	Scan type	Pixel size (μm)	Voltage (keV/W)	Exp. time (s)	Intensity
30	s‐10	Overview	1.4913	80/7	1.4	5200–6500
36	s‐18	Overview	1.4908	80/7	1.5	5000–7000
48	s‐28	Overview	1.4907	80/7	1.8	5000–8000
54	s‐32	Overview	1.4907	80/7	1.7	5000–7500
60	s‐35	Overview	1.4908	80/7	1.4	5000–6500
80	4‐Ovl	Overview	1.9688	40/3	7	5000–7500
100	3‐Ovl	Overview	1.9714	40/3	8	5000–8300
135	2‐Ovl	Overview	2.19	40/3	4	5000–9000
150	1‐Ovl	Overview	2.0643	40/3	4.5	5000–10,000
165		Overview	2.0635	40/3	4	5000–10,000
185	Pec‐bud	Overview	1.971	80/7	0.8	5500–8200
210	High‐pec	Overview	3.5202	40/3	1.6	5000–10,000
235	Long‐pec	Overview	3.8545	40/3	1.4	5000–10,000
260		Overview	1.9707	80/7	0.8	5300–7500
330	Pec‐fin	Overview	3.5214	40/3	1.5	6000–10,000
80	4‐Ovl	Head detail	0.99801	40/3	20	5000–5700
100	3‐Ovl	Head detail	0.99916	40/3	24	5000–6200
165		Head detail	0.9765	40/3	17	5000–8500
185	Pec‐bud	Head detail	0.9989	80/7	3	5000–6300
210	High‐pec	Head detail	1.4582	40/3	9.5	5000–10,000
235	Long‐pec	Head detail	1.4299	40/3	8.5	5000–10,000
260		Head detail	1.4782	80/7	1.3	5200–7700
330	Pec‐fin	Head detail	1.3727	40/3	9	5000–8500

*Note*: Intensity is the "light" intensity that reaches the detector camera. Typically, the exposure time was set so that the intensity was at least 5000 in the darker parts of the sample.

Abbreviations: exp, exposure; hpf, hours post fertilization; s, second.

The raw data for 3‐D imaging of the samples were acquired using an Xradia 520 Versa 3‐D X‐ray microscope (Zeiss). The X‐ray source was set to 80/7 or 40/3 (keV/W). A thin LE1 filter was used to avoid beam hardening artifacts. To obtain high resolution images, a CCD optical objective (4×) was used. The acquisition parameters were set according to the developmental stage of the sample stages (Table 2). The isotropic voxel size for overview scanning of the whole embryo was 2–3 μm. For detailed scanning of the head region, the isotropic voxel size was set to <1.5 μm. Each sample was rotated 180 + fan degrees along the anterior–posterior (AP) axis. The projection images acquired were checked for sample drifting then reconstructed if of acceptable quality.

**TABLE 2:** Developmental stages and selected characters proposed in this study for the rosy bitterling (*Rhodeus ocellatus*) and a comparison with the zebrafish (*Danio rerio*)

Stage name (*R*. *ocellatus*)	hpf	Staging characters	Stage name (*D*. *rerio*)	hpf
Zygote period				
1‐cell	0.4	Blastodisk appears	1‐Cell	0
Cleavage period				
2‐cell	1.3	First cleavage division	2‐Cell	0.75
Blastula period				
Blastula	3.7	Blastoderm forms, irregular tiers of blastomeres	256‐Cell	2.5
Gastrula period				
50%‐epiboly	15	Shield visible	Shield	6
90%‐epiboly	22.5	Small yolk plug	90% epiboly	9
Convergent	23.5	Yolk plug closure	Bud	10
Neurula period				
Extension	23.8	Yolk plug totally disappeared	Bud	10
Migration	24.2	Head region at the midpoint of the A–V axis	Bud	10
1‐Somite	24.5	Ventral yolk constriction, first somite furrow		
3‐Somite	25.5	Discernible optic primordium	3‐Somite	11
6‐Somite	27	Small dorsal yolk sac extensions (YSEs), hatching	6‐Somite	12
Somitogenesis period				
10‐somite	30	EL = 2.6–2.7 mm, Neural tube, Somite number 10–12, otic placode	10‐Somite	14
18‐somite	36	EL = 2.8–3.1 mm, roll‐like tail bud protrusion, somite number 13–20, chevron‐shaped trunk somites, optic vesicle, trigeminal placode, pronephric duct	14‐Somite	16
28‐somite	48	EL = 3.2–3.3 mm, tail elongate, somite number 19–29, heart tube, muscular twitches of skeletal muscles, optic cup, lens placode, otic vesicle, cephalic flexure, rhombomeres	18‐Somite	18
32‐somite	54	EL = 3.6–3.8 mm, somite number 30–32, otolith, optic stalk, heart cone‐shaped, irregular cardiac contraction, nephron primordium, notochord vacuoles, body movements (side‐to‐side flexing)	21‐Somite	19.5
35‐somite	60	EL = 3.7–4.0 mm, tail blade‐shaped, somite number reaches maximum (35), startle response	26‐Somite	22
Pharyngula period				
4‐Ovl	80	EL = 3.9–4.1 mm, lens formation, median fin fold, blood islands, tubular heart, cloaca		
3‐Ovl	100	EL = 4.5–4.7 mm, prim‐6, red blood cells circulating, telencephalon eversion, heart looping, heartbeat; caudal fin rays, caudal vein plexus	Prim‐5	24
2‐Ovl	135	EL = 4.8–4.9 mm, prim‐10, pituitary, heart forward, heartbeat rhythmic, aortic arch, ventral aorta, thickened otic vesicle wall, pectoral fin bud primordium, gut a solid endodermal rod	Prim‐10	~27
1‐Ovl	150	EL = 5.1 mm, prim‐20 to 24, sparse melanophore pigmentation in retina, low pectoral fin bud, semicircular canals, YSEs reaches maximal size	Prim‐25	36
Organogenetic period				
Pec‐bud	185	EL = 5.5 mm, head straightening, olfactory bulb, AER of pectoral fin bud, branchial arches, dorsal body pigmentation, regionalization of median fin fold into dorsal and ventral fins, liver tissue appears		
High‐pec	210	EL = 5.7 mm, lower jaw recognizable, cartilage in mandibular and hyoid arches, pericardium cavity, heart divided into atrium and ventricle	High‐pec	42
Long‐pec	235	EL = 5.8–6.0 mm, head pigmentation, membranous pectoral fin, dorsal fin primordium, mouth open at ventral side, pharyngeal teeth, operculum, gill slits open, gill filaments, gall bladder	Long‐pec	48
Pec‐fin	330	EL = 6.1–6.3 mm, iridophores in iris, YSEs regressing, jaw protruding, mouth opening rostrally, caudal fin rays, melanophores in lateral stripes, three pairs of otoliths, swim bladder	Pec‐fin	60

*Note*: The stages for the zebrafish are taken from Kimmel et al. (1995).

Abbreviations: A–V, animal–vegetal; AER, apical ectodermal ridge; EL, standard embryo length from rostral to caudal (including the tail); hpf, hours post fertilization; Ovl, otic vesicle length.

### 2.5 Image processing

Reconstructed tomographic data for each sample were 3‐D rendered using Avizo software (Version: 9.5.0; Thermo Fisher Scientific), and further processed for viewing in coronal, sagittal and transverse section planes. The 3‐D views were pseudo‐colored with the volume rendering module using physics.icol as the colormap in the Avizo software. Annotations were applied to the virtual sections using Adobe InDesign software (Version: 15.0.2, Adobe Systems Inc., San José, CA). For complex structures (e.g., the semicircular canals of the inner ear), the segmentation of a region of interest was performed in Avizo from the reconstructed images, in order to verify the annotations. Anatomical terms, including those used in the annotations of the figures, were based on the ZFIN anatomical ontology (https://zfin.org/action/ontology/search, Belmamoune & Verbeek, 2007; van Slyke et al., 2014).

## 3 RESULTS

*Notes*: In the following descriptions, the abbreviations in parentheses are the same as those used in the figure annotations. The developmental age of each stage is recorded as hours post fertilization (hpf) at an incubation temperature of 22.5°C. The references to zebrafish stages are to Kimmel et al. (1995); we shall refer to them as “Kimmel” stages. Please note that there is (i) a single *yolk extension* (the yolk extension caudally to the yolk constriction) and (ii) a pair of *yolk sac extensions* (YSEs) dorsolateral to the yolk ball and mainly composed by the thickening of the yolk sac. "Somite number" refers to the number of pairs of somites.

Our results are divided into: (i) pre‐hatching stages; and (ii) post‐hatching stages (Figure 1). The pre‐hatching stages begin at fertilization, and include cleavage, blastula, gastrula and neurula periods, and end at hatching. These pre‐hatching stages were all studied by time‐lapse videography in live embryos. The developmental age was calculated from the time‐lapse videos. The post‐hatching stages include the somitogenesis, pharyngula, and organogenetic periods. These periods were originally applied to zebrafish development by Kimmel et al. (1995). The definition of these “periods” is arbitrary, but useful for organizing the stages and comparative with zebrafish staging series.

### 3.1 Pre‐hatching stages

3.1.1

##### STAGE 1: 1‐cell, 0.4 hpf

**FIGURE 3:**
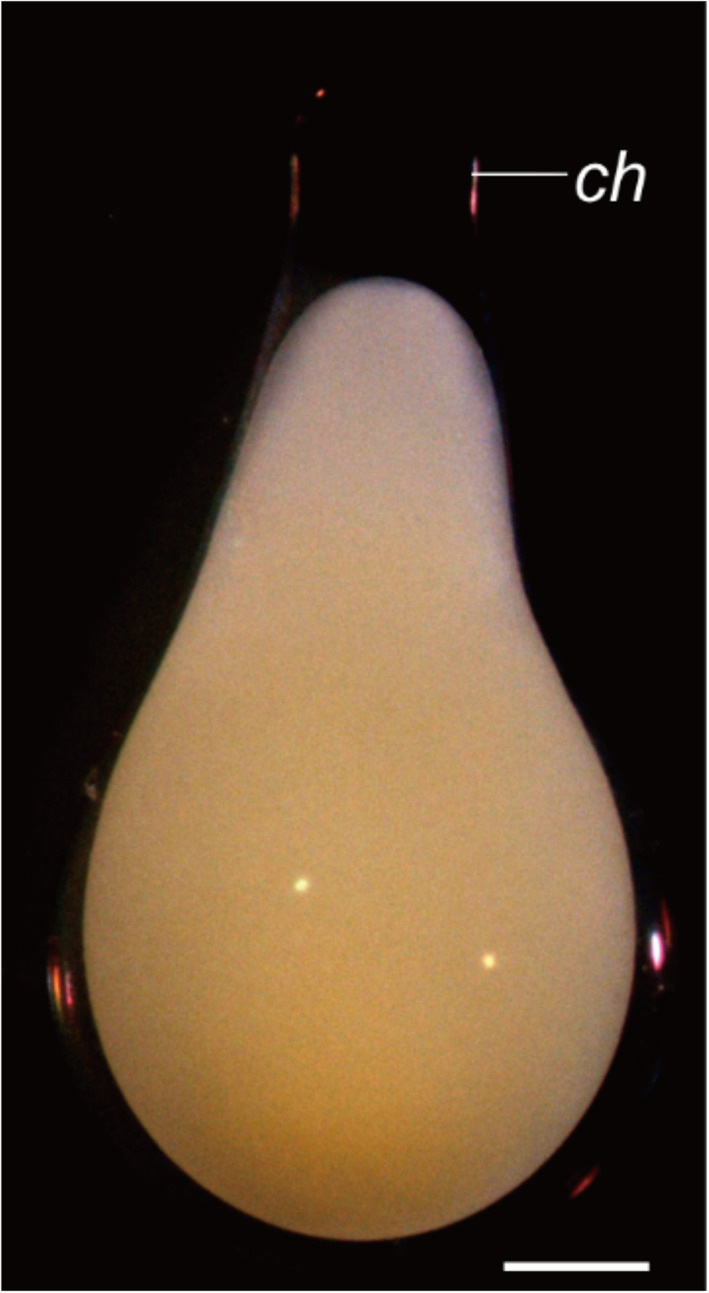
*Rhodeus ocellatus*, zygote period, photomicrograph. The 1‐cell stage zygote within its bulb‐shaped chorion, 20 mins after fertilization. Animal pole to the top; ch, chorion; scale bar = 300 μm

**FIGURE 4:**
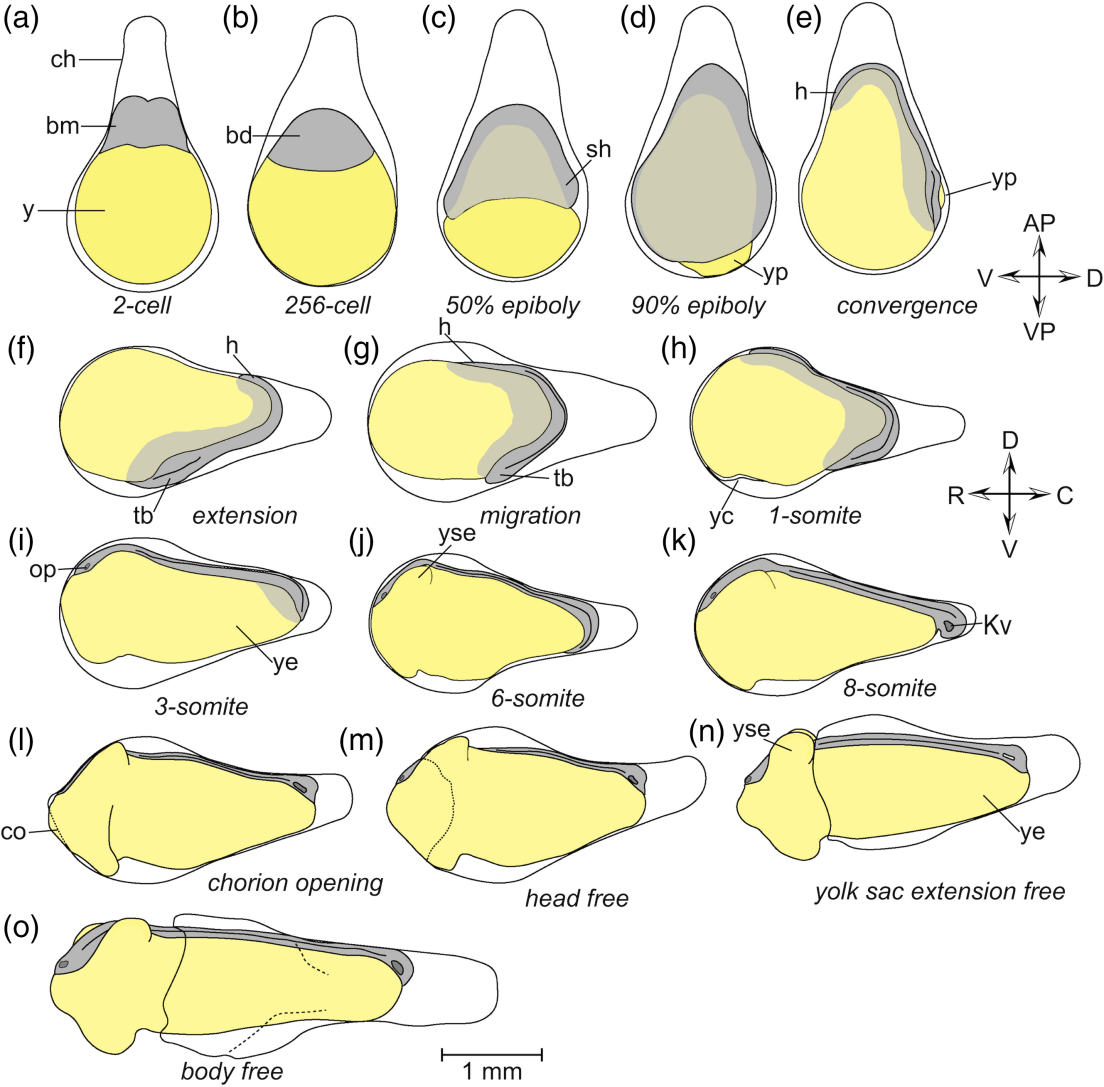
*Rhodeus ocellatus*, pre‐hatching stages, schematic illustration based on time‐lapse photomicrographs. The transparent chorion is represented by a solid line, the ruptured chorion by a dashed line. The blastoderm, and the tissue derived from it, are shaded in light and dark gray, respectively. The yolk is shown in yellow. a–e are lateral views, with the animal pole at top, ventral to the left. In f–o, the lateral view is rotated 90° clockwise (relative to a–e), with dorsal to the top, rostral to the left. Abbreviations: AP, animal pole; bd, blastoderm; bm, blastomere; C, caudal; Ch, chorion; co, chorion opening; D, dorsal; h, head; Kv, Kupffer's vesicle; op, optic primordium; R, rostral; sh, shield; tb, tail bud; V, ventral; VP, vegetal pole; y, yolk; yc, yolk constriction; ye, yolk extension; yp, yolk plug; yse, yolk sac extension

The eggs of *R*. *ocellatus* are demersal (inclined to sink in water). The chorion is bulb‐shaped, with an elongated stalk at the animal pole (Figure 3). The perivitelline space between the embryo and the chorion forms as the latter swells and lifts away from the embryo; it is narrow at the vegetal pole. Activated by fertilization, the yolk‐poor cytoplasm streams towards the animal pole (AP), forming the blastodisk. By contrast, the yolky cytoplasm remains at the vegetal pole (VP) forming the yolk ball (Figure 4a). This stage is comparable to Kimmel stage *1‐cell*.

##### STAGE 2: 2‐cell, 1.3 hpf

Cleavage is meroblastic, as in other teleosts including *Danio rerio* (Kimmel et al., 1995). At the 2‐cell stage, the blastodisk becomes divided symmetrically, forming two equally‐sized blastomeres (Figure 4a). This stage is comparable to Kimmel stage *2‐cell*.

##### STAGE 3: Blastula, 3.7 hpf

**FIGURE 5:**
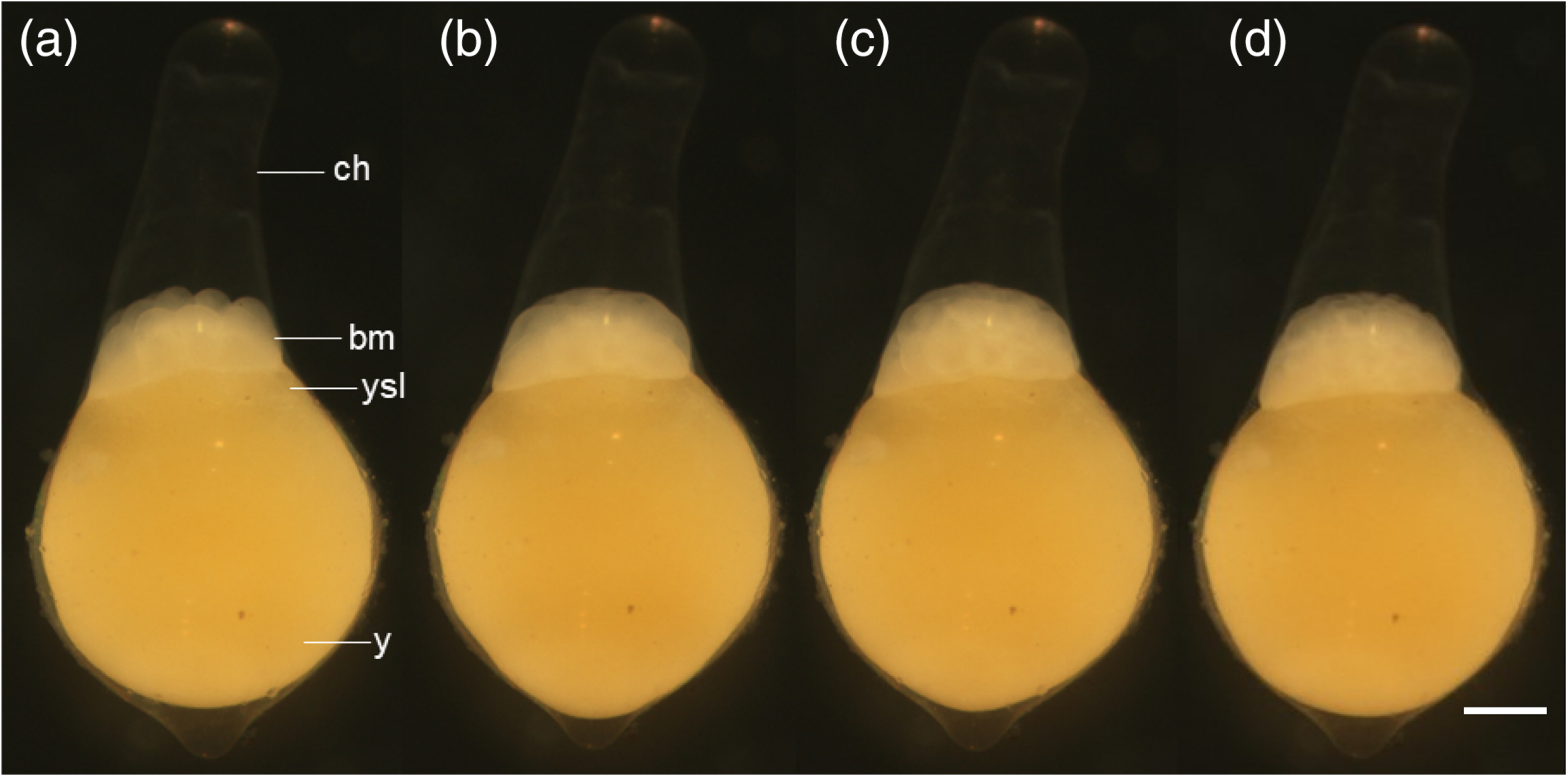
*Rhodeus ocellatus*, embryos during the cleavage period, photomicrographs. Animal pole to the top. (a) 16‐cell stage (1.75 hpf). (b) 32‐cell stage (2.2 hpf). (c) 64‐cell stage (2.65 hpf). (d) 128‐cell stage (3.1 hpf). Abbreviations: bm, blastomere; ch, chorion; y, yolk; ysl, yolk syncytial layer. Scale bar = 300 μm

The blastula stage (Figures 4b and 5) is characterized by the proliferation of blastomeres so that they come to form cells of many layers deep. A distinct border, the yolk syncytial layer (YSL), appears between the blastodisk and yolk. In late blastula stages, epiboly movements start so that the blastodisk spreads towards the vegetal pole, engulfing the underlying yolk ball. The animal–vegetal (A–V) axis becomes shortened and the shape of the embryo changes from pear‐shape to ellipsoid (compare Figure 4a,b). This stage is comparable to Kimmel stage *256‐cell*.

##### STAGE 4: 50% epiboly, 15 hpf

**FIGURE 6:**
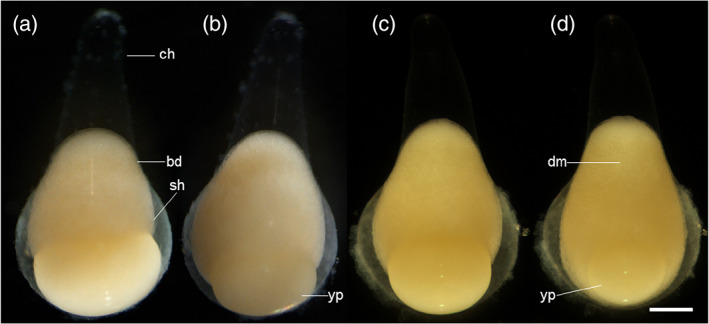
*Rhodeus ocellatus*, embryos during the epiboly period, photomicrographs. (a and b) left view, animal pole to the top, dorsal to the left. (a) 60% epiboly stage (16 hpf). (b) 80% epiboly stage (20 hpf). (C and d) dorsal view. (c) 80% epiboly stage (20 hpf). (d) 90% epiboly stage (22.5 hpf). Abbreviations: bd, blastoderm; ch, chorion; dm, dorsal midline; sh, shield; yp, yolk plug. Scale bar = 400 μm

Epiboly is coordinated by three morphogenetic movements: spreading, convergence, and extension (Xiong et al., 2014). First, the blastoderm covers the yolk by gradually spreading over it. The progress of epiboly can be expressed in terms of the percentage coverage of the yolk ball by the blastodisk margin. At the beginning of epiboly, the blastoderm is uniform in thickness. Later, the spreading cells converge on the dorsal midline of the embryo and the embryonic shield is thereby formed (Figures 4c and 6a). After formation of the shield, the dorsoventral (D‐V) and the rostrocaudal (R‐C) axes are distinct. The shield is now at the caudal end of the dorsal midline. This stage is comparable to Kimmel stage *shield*.

##### STAGE 5: 90% epiboly, 22.5 hpf

**FIGURE 7:**
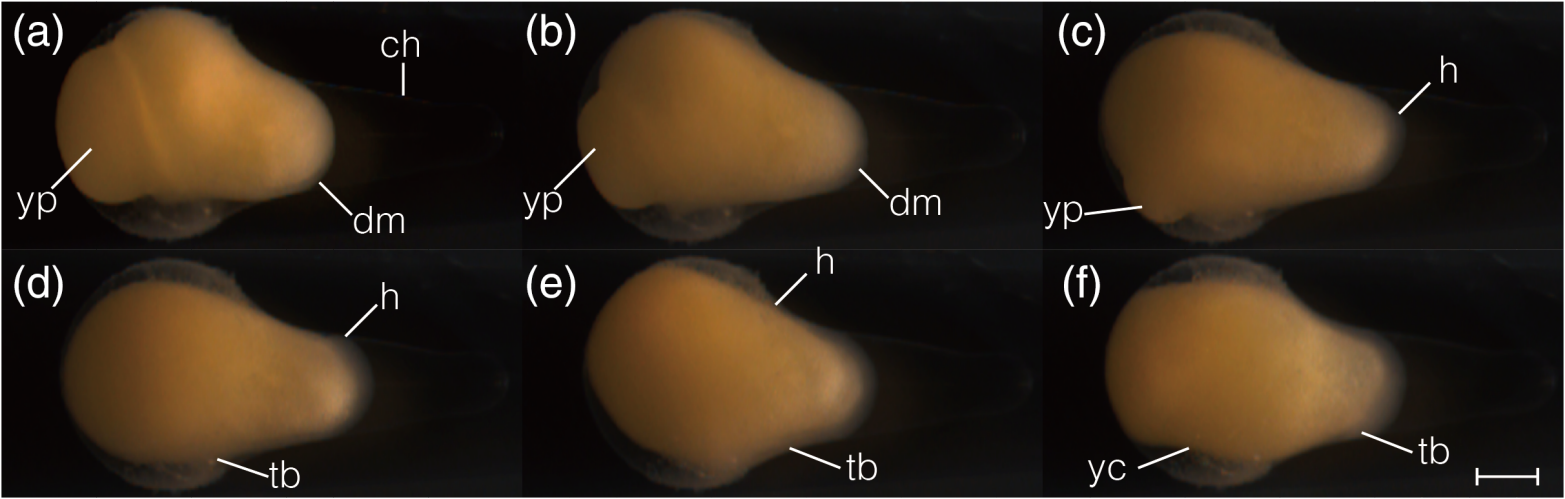
*Rhodeus ocellatus*, embryos from yolk‐plug closure to tail bud present, photomicrographs. Lateral views. The position of embryo is changing so that, at the final stage (f) dorsal is at the top, rostral to the left. (a) 80% epiboly stage (20 hpf), embryo with a large yolk‐plug at the vegetal pole. (b) 90% epiboly stage (22.5 hpf), embryonic tissue condensed along the dorsal midline. (c) end of epiboly stage (23.5 hpf), embryo with a small yolk plug. The developing head region is at the animal pole, with dorsal to the top, rostral to the left; (d) neurula extension stage (23.8 hpf), yolk plug has disappeared, tail bud present, head region extents towards ventral side. (e) neurula migration stage (24.2 hpf), head migrates towards the vegetal pole. (f) 1‐somite stage (24.5 hpf), yolk constriction appears at the ventral side. Abbreviations: AP, animal pole; ch, chorion; dm, dorsal midline; h, head; tb, tail bud; VP, vegetal pole; yc, yolk constriction; yp, yolk plug. Scale bar = 500 μm

The margin of the blastoderm is no longer perpendicular to the A–V axis; the ventral part has spread further than the more compact dorsal part. Therefore, the yolk plug is not located precisely at the vegetal pole but on the dorsal side of the A–V axis (Figures 4D, 6D, and 7B). Comparable to Kimmel stage *90% Epiboly*.

##### STAGE 6: Convergence, 23.5 hpf

A small yolk plug is present on the dorsal side of the A–V axis (Figures 4e and 7c); epiboly ends when the yolk plug disappears. The developing head region is becoming visible as a cellular condensation near the animal pole. The developing neural primordium is visible in the dorsal midline, flanked by on each side by paraxial mesoderm. This stage is comparable to Kimmel stage *bud*.

##### STAGE 7: Neurula extension, 23.8 hpf

Neurula stages (Figures 4f and 7d) begin after closure of the yolk plug. At the neurula extension stage, the head region, notochord rudiment, and tailbud are distinguishable. Neurula extension movements cause the head region to become displaced towards the vegetal pole. The head is located near the narrowed stalk part of the chorion. This stage is comparable to Kimmel stage *bud*.

##### STAGE 8: Neurula migration, 24.2 hpf

The head region extends along the R‐C axis towards the widened part of the chorion. In time‐lapse movies, it appears as though the embryonic tissue migrates on the yolk ball surface, driven by the neurula extension movements, and we therefore name this stage the “neurula migration” stage (Figures 4g and 7e). This stage is comparable to Kimmel stage *bud*.

##### STAGE 9: 1‐somite, 24.5 hpf

The first somitic furrow appears. Somitogenesis (Figures 4h and 7f) overlaps in time with neurulation. The intersomitic boundaries are not easily discernible under the dissection microscope. Therefore, yolk shape can also be used to define this stage. The yolk constriction appears at this stage on the ventral side of the embryo and deepens towards the dorsal side. This stage is intermediate between Kimmel stages *bud* and *3‐somit*

##### STAGE 10: 3‐somite, 25.5 hpf

There are three somite pairs. The head has assumed its definitive location at the end of the wide bulb‐end of the chorion. During neurulation the neural ectoderm develops into the neural plate, which forms the neural keel by primary neurulation (Lowery & Sive, 2004). The neural keel is triangular in cross‐section, and initially solid; it later forms the neural rod which has a circular cross‐section and is also solid. The eye field, a common primordium of both the left and right eyes (Figure 4i), is the only discernible sensory primordium at this stage. Comparable to Kimmel stage *3‐som*

##### STAGE 11: 6‐somite, 27 hpf

There are six somite pairs. A pair of yolk sac extensions (YSEs) forms ventrolaterally on the trunk. Kupffer's vesicle appears on the ventral aspect of the tailbud just before hatching (Figure 4j,k). Hatching (Figure 4l–o) does not take place at a consistent developmental age and is therefore not part of our series of named stages. The typical time of hatching that we observed here was 28 hpf – 35 hpf. The initial rupture is always in the “bulb” of the chorion (Figure 4l), and the rostrally protruded yolk comes to protrude from that opening. As the opening becomes enlarged, the head region of the embryo also emerges from the chorion (Figure 4m). Then, the widest part of the embryo, that is the region of the YSE, also emerges from the opening in the chorion (Figure 4n). By now, the posterior part of the embryo is still inside the chorion; gradually however, the entire chorion is pushed away by the increasing length of the body and elongation of tail, and by the intermittent side‐to‐side movements of the tail observed in videography (Figure 4o). Comparable to Kimmel stage *6‐somite*.

### 3.2 Post‐hatching stages

Post‐hatching stages are divided into somitogenesis, pharyngula, and organogenetic periods.

#### 3.2.1 Segmentation period

**FIGURE 8:**
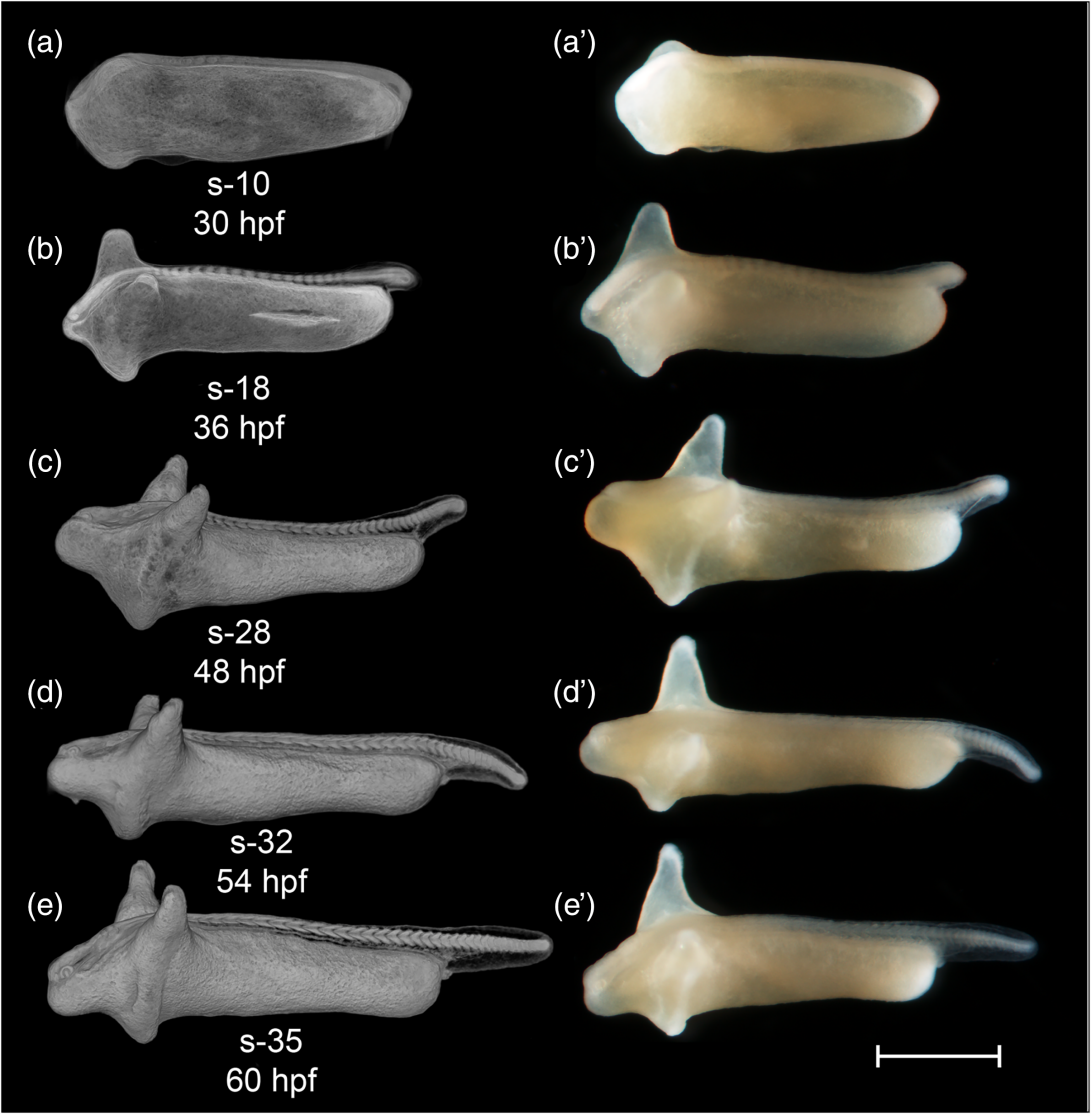
*Rhodeus ocellatus*, stages during the somitogenesis period. (a–e) microCT images, volume rendering. (a'–e') photomicrographs of the same embryo. (a and a') Stage 10‐somite, 30 hpf. (b and b') Stage 18‐somite, 36 hpf. (c and c') Stage 28‐somite, 42 hpf. (d and d') Stage 32‐somite, 54 hpf. (e and e') Stage 35‐somite, 60 hpf. Lateral view, dorsal up, head to the left. Abbreviations: Hpf, hours post‐fertilization; s, somite. Scale bar = 1 mm

During the somitogenesis period, segmentation of somites continues and rhombomeres develop. The somite number is a quantal (discrete) staging character and is therefore particularly useful in comparative developmental studies (Battle, 1940; Furutani‐Seiki & Wittbrodt, 2004; Iwamatsu, 2004; Signore et al., 2009; Tsai et al., 2013). The elongation of the tail during the somitogenesis period is a useful staging character. Embryos before tailbud protrusion have <12 somite pairs (Figure 8a). An elongated, cylindrical tailbud indicates that somitogenesis in the truncal region is complete and there are 12–22 somites (Figure 8b). As the tailbud elongates further, caudal somites develop and reach the final number of 35 (Figure 8c,d,e).

##### STAGE 12: 10‐somite, 30 hpf

**FIGURE 9:**
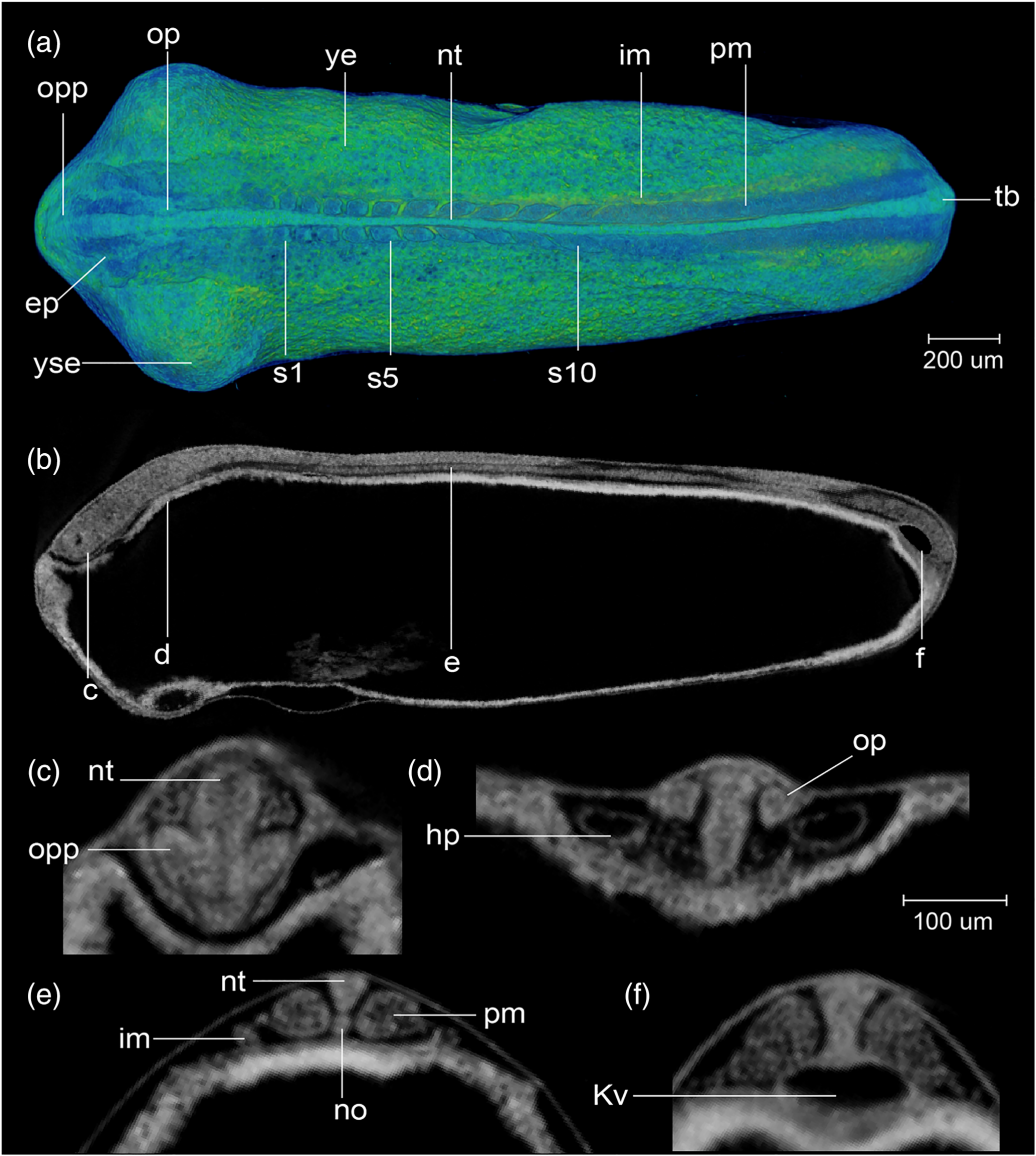
*Rhodeus ocellatus*, stage 10‐somite, microCT images. (a) Volume rendering, dorsal view, rostral to the left. (b) Virtual section, midsagittal, dorsal to the top, rostral left, letters C–F indicates the section level of the correspondent transverse sections. (c–e) Virtual sections, transverse, dorsal to the top. Abbreviations: ep, ectoderm placode; hp, heart primordium; im, intermediate mesoderm; Kv, Kupffer's vesicle; no, notochord; nt, neural tube; op, otic placode; opp, optic primordium; pm, paraxial mesoderm; s, somite; tb, tail bud; ye, yolk extension; yse, yolk sac extension

There are 10 somite pairs (Figure 9a). The somites are followed caudally by unsegmented paraxial mesoderm. Intermediate mesoderm is discernible as two rods at the ventrolateral edge of the somitic mesoderm (Figure 9a,e). The tailbud is not yet free from the yolk (Figure 8a,a'). Kupffer's vesicle is still recognizable ventral to the tailbud (Figures 8a and 9f). The yolk constriction is prominent on the ventral aspect of the yolk, near the head end of the embryo (Figure 8a). The neural tube is differentiated into a brain primordium and narrower spinal cord primordium (Figure 9a,c). The optic primordia extend laterally from the future diencephalon so that the outline of the head in dorsal view is arrowhead shaped (Figure 9a,c). Ectodermal placodes first become visible with microCT as thickenings in the ectoderm lateral to the neural tube (Figure 9a). The otic placodes are located midway between the optic primordia and the first somite (Figure 9a,d). There are paired heart primordia in the splanchnic mesoderm (Figure 9d). Most embryos have hatched completely, but in a few cases, the posterior part of the body is still enclosed. This stage is comparable to Kimmel stage *10‐somite*.

##### STAGE 13: 18‐somite, 36 hpf

**FIGURE 10:**
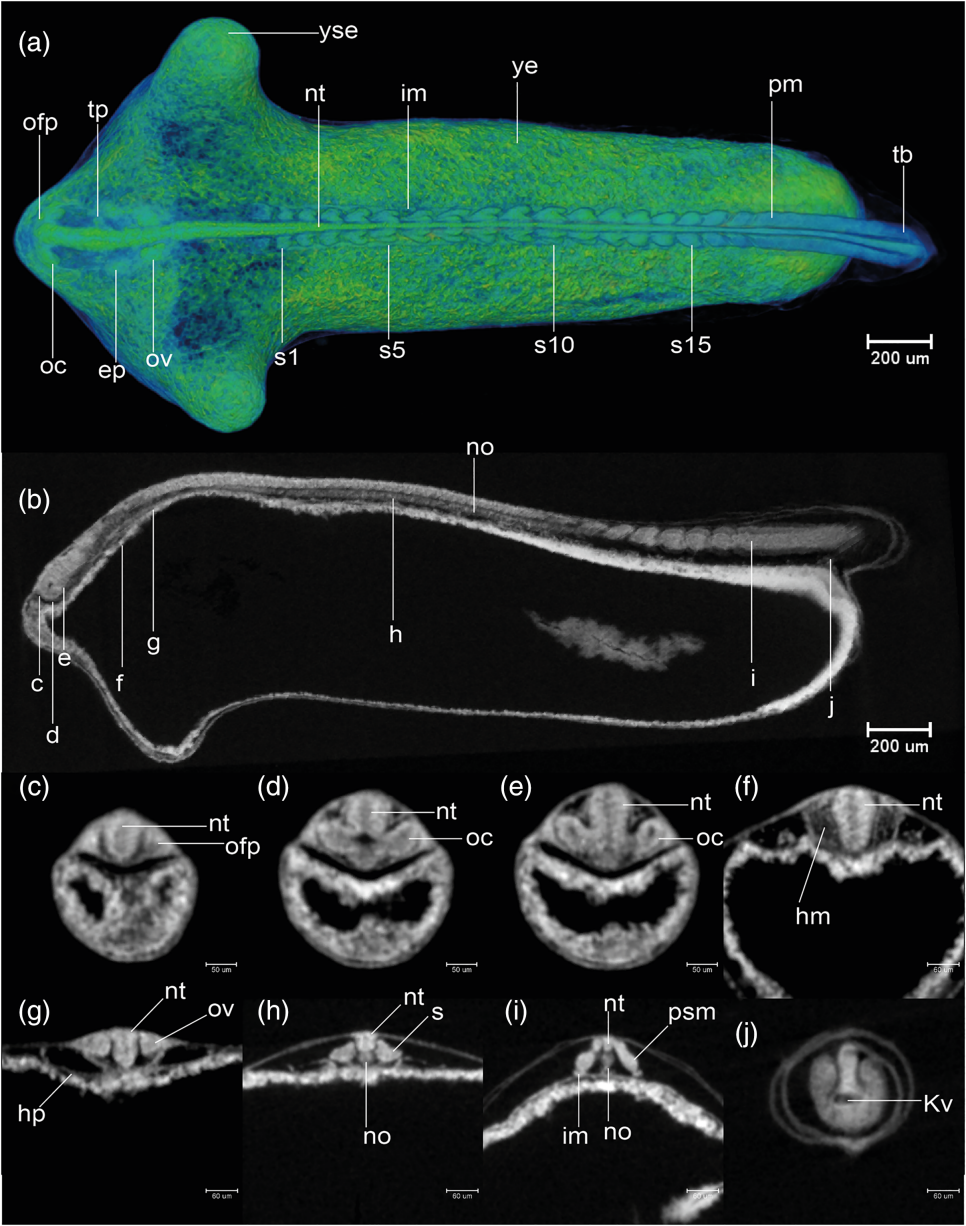
*Rhodeus ocellatus*, stage 18‐somite, microCT images. (a) Volume rendering, dorsal view, rostral to the left. (b) Virtual section, midsagittal, dorsal to the top, rostral left, letters C–J indicates the section level of the correspondent transverse sections. (c–j) Virtual sections, transverse, dorsal to the top. Abbreviations: ep, ectoderm placode; hm, head mesoderm; hp, heart primordium; im, intermediate mesoderm; Kv, Kupffer's vesicle; no, notochord; nt, neural tube; oc, optocoele; ofp, olfactory placode; ov, otic vesicle; pm, paraxial mesoderm; psm, presomitic mesoderm; s, somite; t, tail; tg, trigeminal placode; ye, yolk extension; yse, yolk sac extension

**FIGURE 11:**
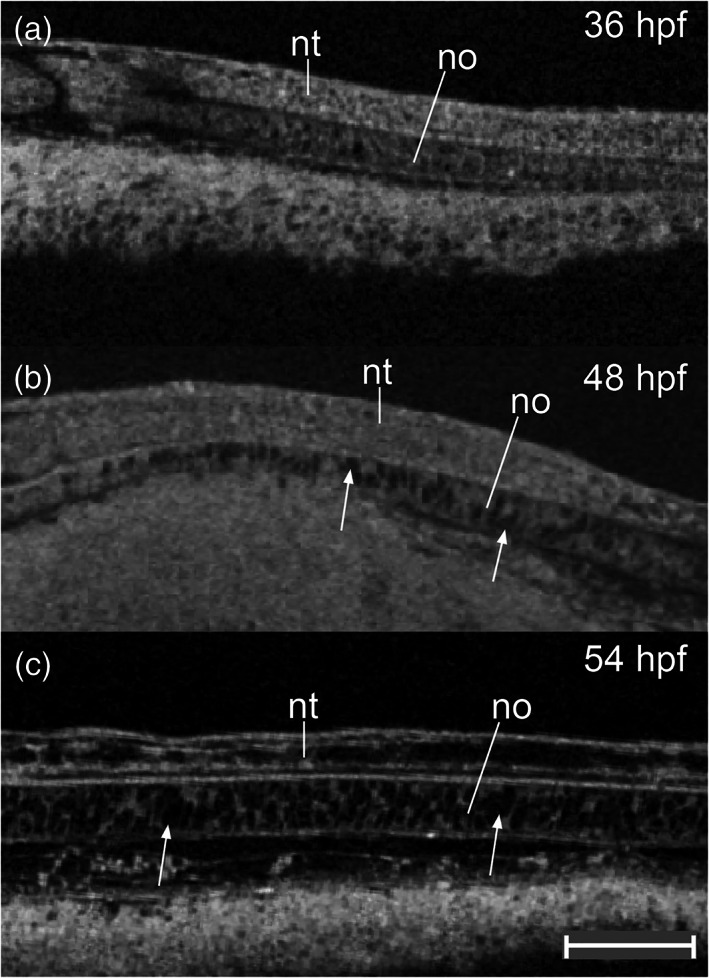
*Rhodeus ocellatus*, development of the notochord, microCT images, virtual sections. (a–c) Sagittal view, dorsal to the top, rostral left. (a) Stage 18‐somite, 36 hpf. (b) Stage 28‐somite, 48 hpf. (c) Stage 32‐somite, 54 hpf. Abbreviations: no, notochord; nt, neural tube. Vacuolated cells are indicated by arrowheads. Scale bar = 100 μm

There are 18 somite pairs (Figure 10a). The first and last three somites are cuboidal in shape, whereas the remaining somites have the characteristic chevron‐shape of the adult myotome (Figure 10a). The tailbud now projects beyond the yolk, on the dorsal side, forming a cylindrical protrusion (Figures 8b and 10a). Kupffer's vesicle is now decreasing in size. It is now located close to the tip of the tailbud (Figure 10j). The yolk extension is elongated posteriorly. The YSEs project dorsally (Figures 8b and 10a). The neural tube is now cylindrical in transverse section and has a lumen (compare Figure 10d,e). The optic vesicle now has a lumen (optocoele) but is not yet invaginated into a cup (Figure 10d). The otic placodes are condensed, but lack a lumen at this stage (Figure 10g). The trigeminal placodes are present midway between the optic vesicle and otic placode, adjacent to the future rhombomere 2, and posterior to the nascent midbrain–hindbrain boundary (Figure 10a). The cardiac primordia are visible as a pair of hollow tubes (Figure 10g). The notochord has stack‐of‐coins appearance (Figure 11a). A pair of pronephric ducts is seen, one on each side of the dorsal aorta (Figure 10i). This stage is comparable to Kimmel stages *14‐somite* and *18‐somite*.

##### STAGE 14: 28‐somite, 48 hpf (2 dpf)

**FIGURE 12:**
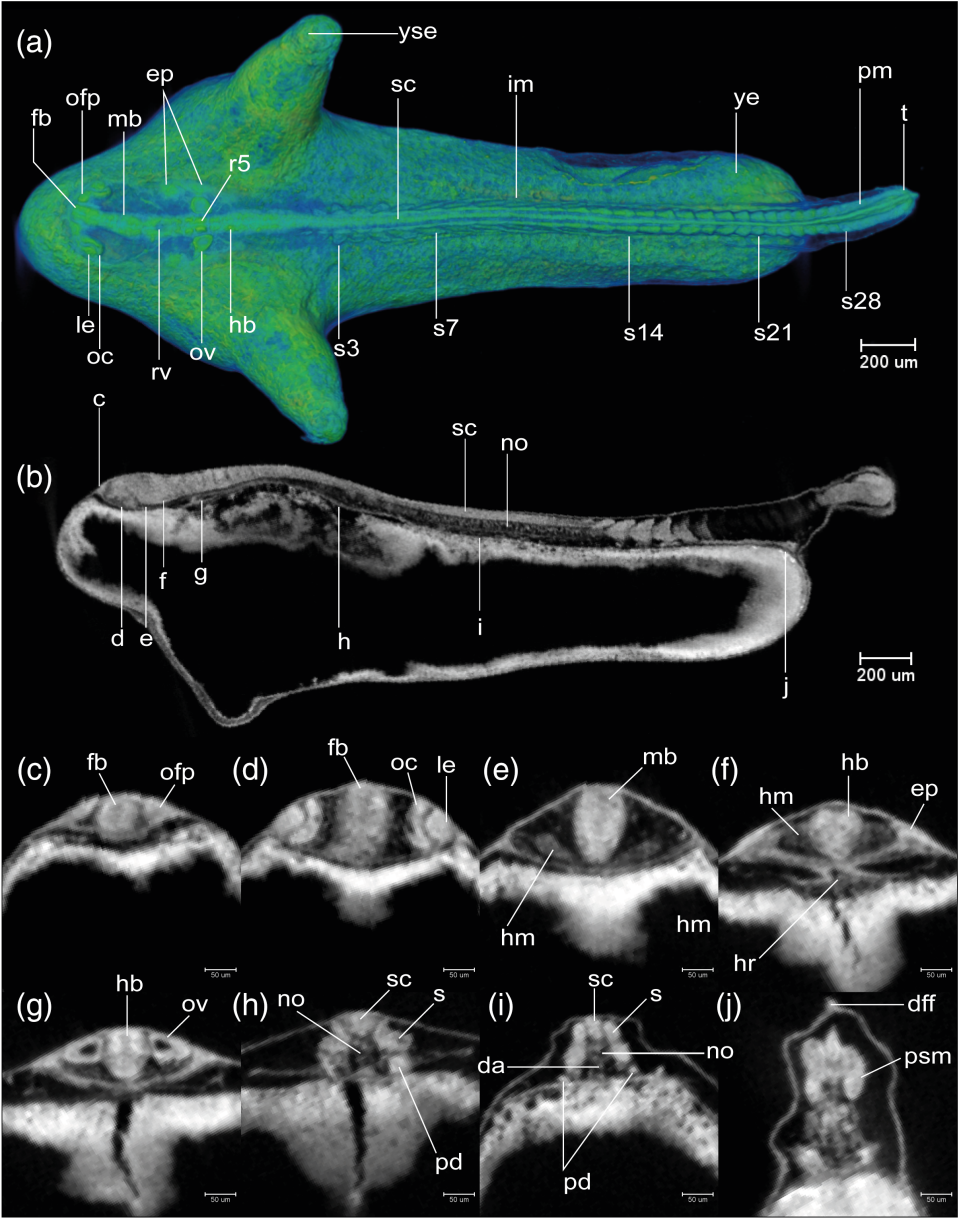
*Rhodeus ocellatus*, stage 28‐somite, microCT images. (a) Volume rendering, dorsal view, rostral to the left. (b) Virtual section, midsagittal, dorsal to the top, rostral left, letters C–J indicates the section level of the correspondent transverse sections. (c–j) Virtual sections, transverse, dorsal up. Abbreviations: da, dorsal aorta; dff, dorsal part of median fin fold, ep, ectoderm placode; fb, forebrain; hb, hindbrain; hm, head mesoderm; hr, heart; im, intermediate mesoderm; le, lens; mb, midbrain; no, notochord; nt, neural tube; oc, optic cup; ofp, olfactory placode; ov, otic vesicle; rv, rhombencephalic ventricle; pd, pronephric duct; pm, paraxial mesoderm; psm, presomitic mesoderm; s, somite; sc, spinal cord; t, tail; ye, yolk extension; yse, yolk sac extension

There are 28 somite pairs. The YSEs are increasingly narrowed at their tips (Figures 8c and 12a). Kupffer's vesicle is no longer visible (Figure 12j). The tailbud is flexed dorsally at its caudal end (Figure 8c). The median fin fold is now visible as a continuous ridge extending the length of the tail (Figures 8c and 12j). The cephalic flexure of the neural tube is now apparent, dorsal to the hypothalamus (Figure 8b). The neural tube is completely hollow (Figure 12a). Forebrain, midbrain and hindbrain regions are distinct (Figure 12a). The midbrain–hindbrain boundary (mhb) is s a shallow constriction (isthmus) of the neural tube (Figure 12a). The midbrain and hindbrain ventricles are becoming expanded (Figure 12a). Rhombomeres (neuromeres) 2–6 (r2‐6) are visible as shallow indentations of the neural tube (Figure 12a). The olfactory placodes thicken and appear oval in shape; in dorsal view, they are situated between the forebrain and the eyes (Figure 12a,c). The optic vesicle is now cup‐shaped (Figure 12a,d). The lens placodes appear and are located within the invaginating optic cups (Figure 12d). The otic vesicle is present at the axial level of r5 and is now a hollow vesicle with a simple, ovoid lumen (Figure 12a,g). Otoliths are not yet visible with microCT. Epibranchial placodes are present ventrolateral to the otic vesicles (Figure 12a,f). The heart is conical with its apex directed dorsally. A medial section through the apex of the cone shows the endocardial organ as a cluster of cells (Figure 12f). There is no detectable cardiac constriction. The stack‐of‐cells appearance of the notochord is giving way to a vacuolated appearance (Figure 11b). A pair of nephric rudiment appears, one on each side ventral to somite 3 (Figure 12h). The endoderm is thin superficial to the yolk syncytial layer (Figure 12h). This stage is comparable to Kimmel *18‐somite* stage.

##### STAGE 15: 32‐somite, 54 hpf (2.25 dpf)

**FIGURE 13:**
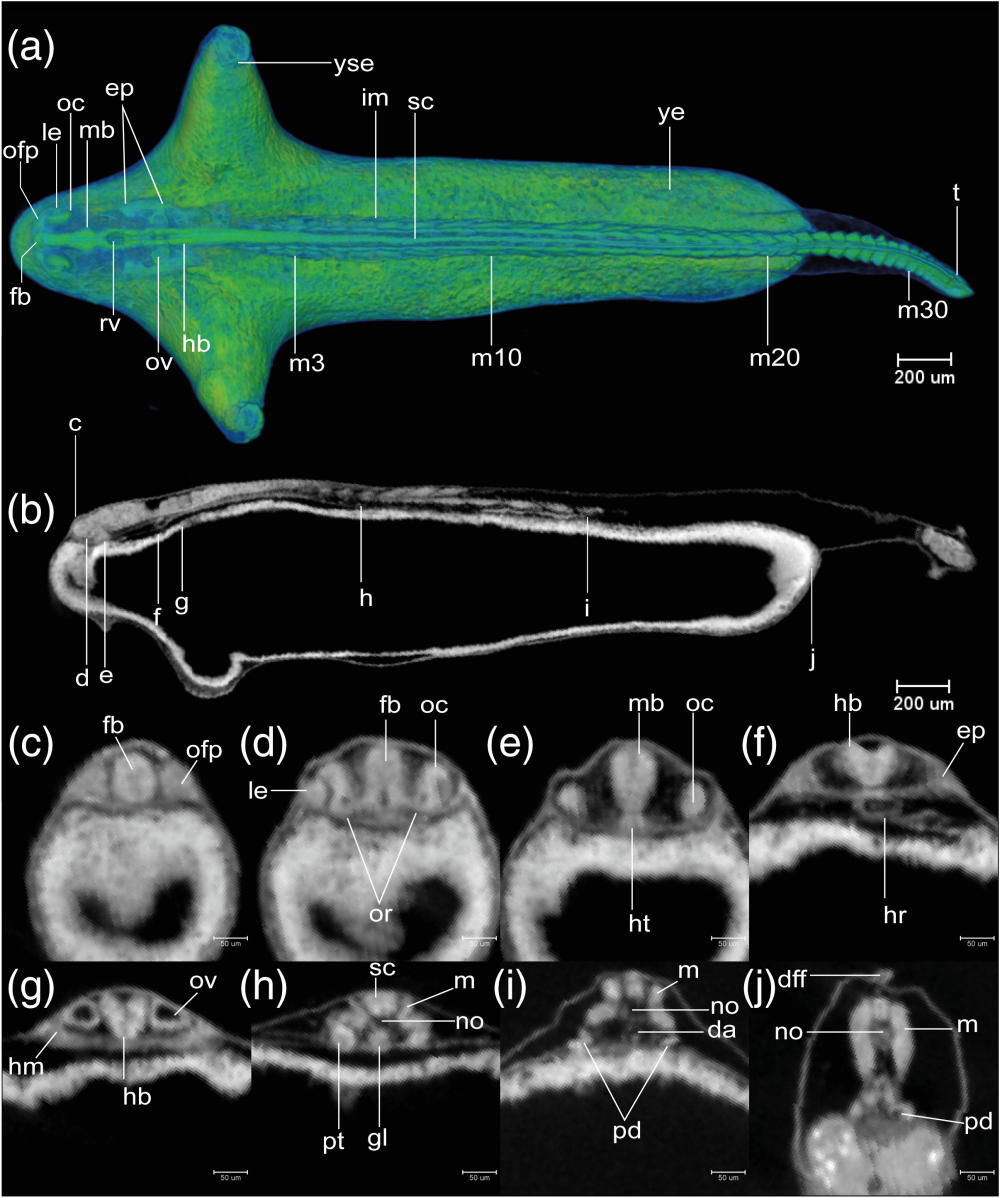
*Rhodeus ocellatus*, stage 32‐somite, microCT images. (a) Volume rendering, dorsal view, rostral to the left. (b) Virtual section, midsagittal, dorsal to the top, rostral left, letters C–J indicates the section level of the correspondent transverse sections. (c–j) Virtual sections, transverse, dorsal up. Abbreviations: da, dorsal aorta; dff, dorsal part of median fin fold; ep, ectoderm placodes; fb, forebrain; gl, pronephric glomerulus; hb, hindbrain; hm, head mesoderm; hr, heart; ht, hypothalamus; le, lens; m, myotome; mb, midbrain; no, notochord; nt, neural tube; oc, optic cup; ofp, olfactory placode; or, optic recess; ov, otic vesicle; rv, rhombencephalic ventricle; pd, pronephric duct; pt, pronephric tubule; sc, spinal cord; t, tail; ye, yolk extension; yse, yolk sac extension

There are 32 somite pairs. The tail tip is flexed ventrally (Figure 8d). The span of the YSEs (the tip‐to‐tip distance) is c. 50% of the rostrocaudal length of the yolk extension (Figure 13a). The optic rudiment is distinctly cup‐shaped (Figure 13a,d). The optic recess (lumen of the optic stalk) is visible at the boundary of telencephalon and diencephalon (Figure 13d). Two pairs of otoliths (the anterior otolith lapillus and the posterior otolith sagitta) are visible in the otic vesicles. Epibranchial placodes and head mesoderm are distinct condensations (Figure 13a,g). The heart is tubular at the axial level of the rhombencephalon (Figure 13f), and exhibits regular peristaltic contraction. At the axial level of somite 3, the nephron primordium has pronephric tubules and pronephric glomeruli (Figure 13). The embryos show irregular spontaneous body movements as the tail thrashes from side‐to‐side. This stage is comparable to Kimmel stage *21‐somite*.

##### STAGE 16: 35‐somite, 60 hpf (2.5 dpf)

**FIGURE 14:**
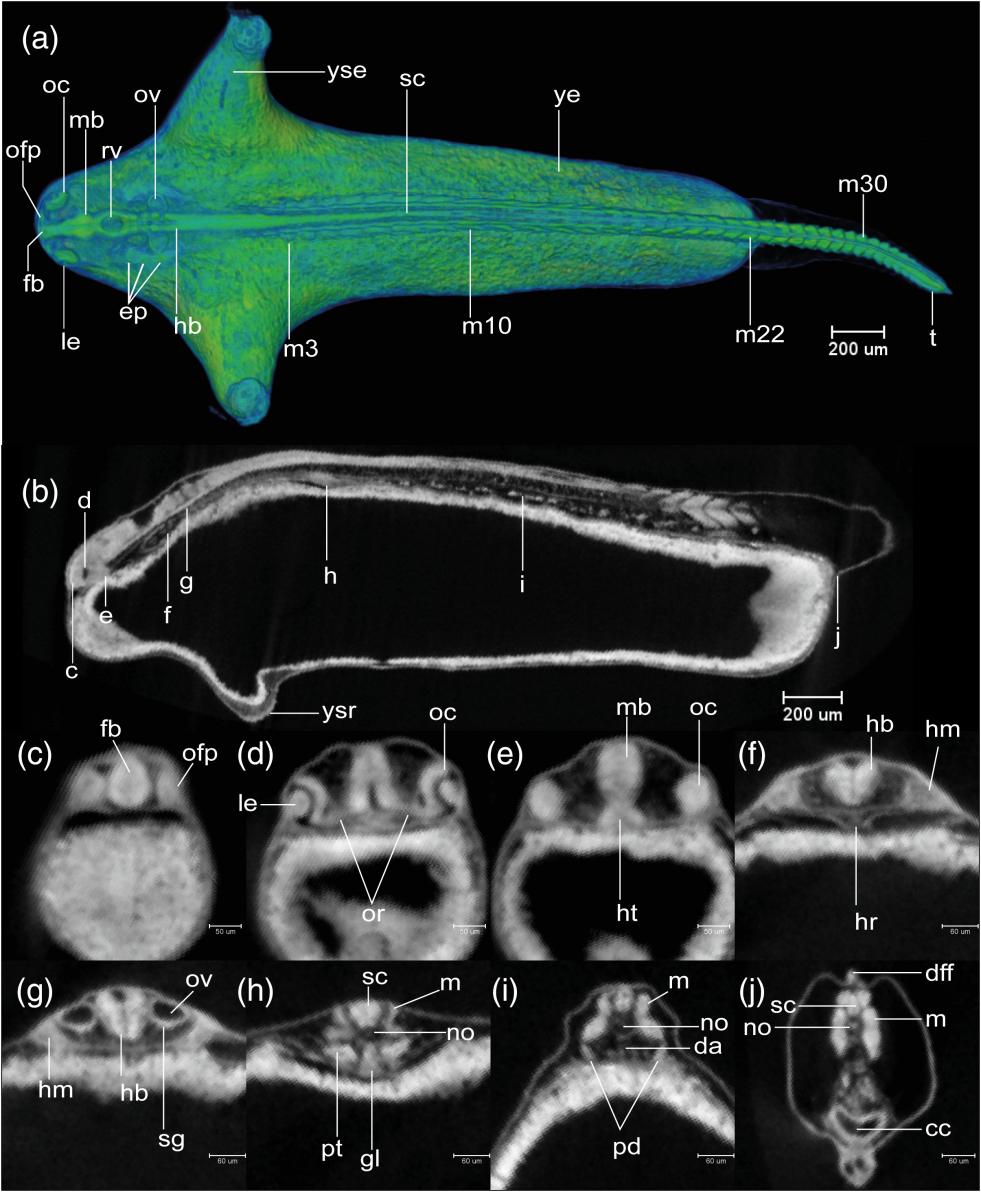
*Rhodeus ocellatus*, stage 35‐somite, microCT images. (a) Volume rendering, dorsal view, rostral to the left. (b) Virtual section, midsagittal, dorsal to the top, rostral left, letters C–J indicates the section level of the correspondent transverse sections. (c–j) Virtual sections, transverse, dorsal up. Abbreviations: cc, cloaca; da, dorsal aorta; dff, dorsal part of median fin fold; ep, ectoderm placodes; fb, forebrain; gl, pronephric glomerulus; hb, hindbrain; hm, head mesoderm; hr, heart; ht, hypothalamus, le, lens; m, myotome; mb, midbrain; no, notochord; oc, optic cup; ofp, olfactory placode; or, optic recess; ov, otic vesicle; rv, rhombencephalic ventricle; pd, pronephric duct; pt, pronephric tubule; sc, spinal cord; sg, rudiment of statoacoustic ganglion; t, tail; ye, yolk extension; yse, yolk sac extension; ysr, yolk sac ridge

The maximum number of 35 pairs is present. The tail is straight and blade‐like (Figure 8e). A continuous ridge extends around the ventral aspect of the yolk sac connecting the two YSEs (Figure 8e). The ventral edge of the yolk sac bulges caudally and forms the yolk sac ridge (Figure 14b). The caudal aspect of this ridge is becoming hollowed‐out (Figures 8e and 14b). As the cephalic flexure increases, ventral bending of the neural axis is more evident (compare Figures 13b and 14b); the hypothalamus and thalamus therefore come to lie ventral to the mesencephalon (Figure 14e). The lens becomes spherical and is partially detached from the surface ectoderm (Figure 14d). The roof of the rhombencephalic ventricle is now thin (Figure 14f,g). The epithelium of the ventral part of the otocyst appears irregular, with cells delaminating as precursors of the statoacoustic ganglion (Figure 14g). The embryos show a touch reflex. This stage is comparable to Kimmel stage *26‐somite*.

#### 3.2.2 Pharyngula period

**FIGURE 15:**
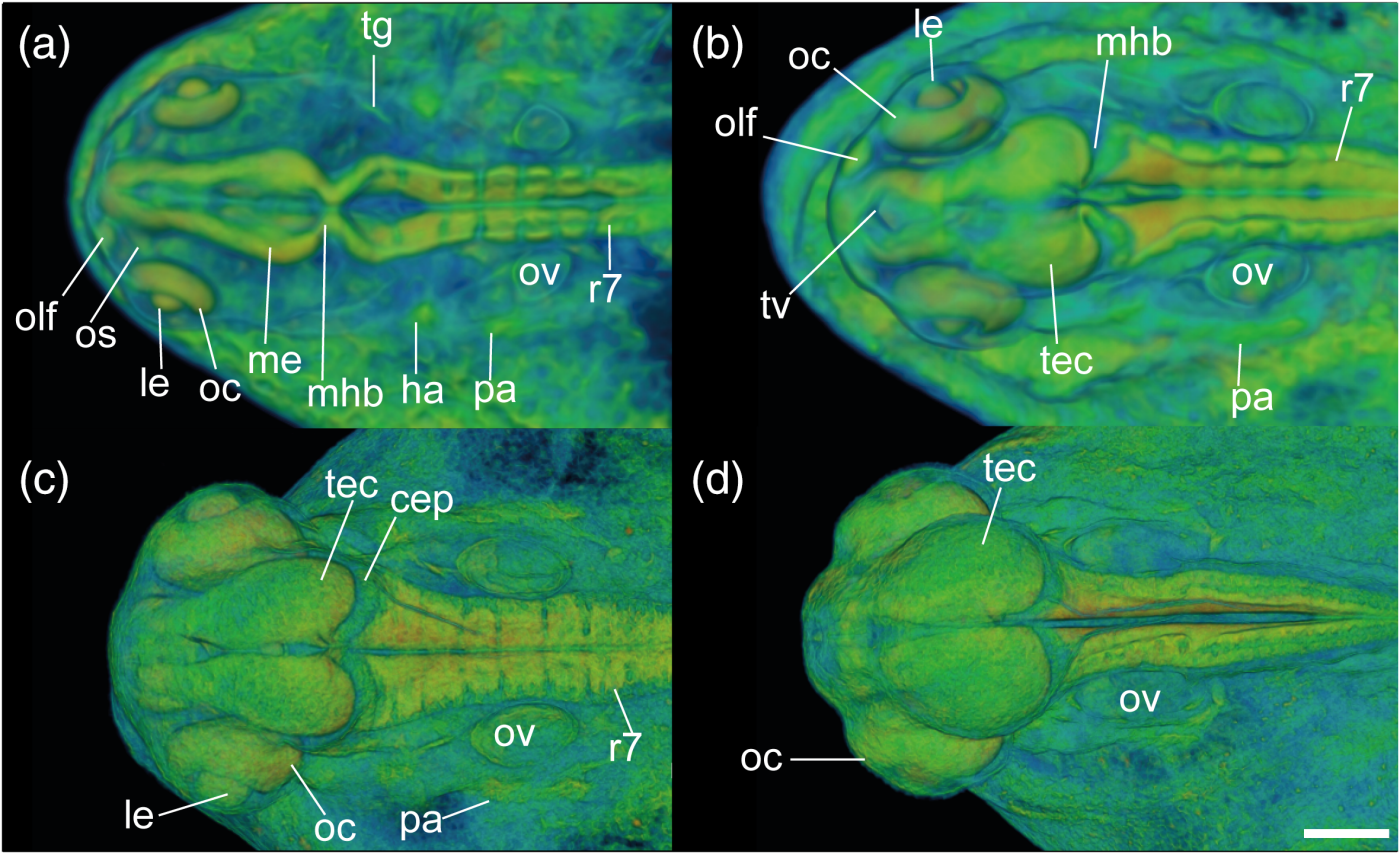
*Rhodeus ocellatus*, embryos during the pharyngula period, microCT images, pseudo‐colored volume‐renderings. Dorsal view, rostral to the left. (a) 4‐ovl, 80 hpf; (b) 3‐ovl, 100 hpf; (c) 2‐ovl, 135 hpf; (d) 1‐ovl, 150 hpf. Abbreviations: cep, cerebellar plate; ha, hyoid arch; le, lens; me, mesencephalon; mhb, midbrain–hindbrain boundary; oc, optic cup; olf, olfactory placode; os, optic stalk; ov, otic vesicle; ovl, otic vesicle length; pa, pharyngeal arch; r, rhombomere; tec, optic tectum; tg, trigeminal ganglion. Scale‐bars, 100 μm in (a), 110 μm in (b) and (c), 130 μm in (d)

In defining this period as the “pharyngula” period, we are following the lead of the Kimmel stages (Kimmel et al., 1995). The term “pharyngula” was introduced by Ballard (1981) to describe a conserved phase of vertebrate development in which the pharyngeal arches are externally visible (Figure 15). During the pharyngula period, we have named each stage using otic vesicle length (ovl), defined by Kimmel as corresponding to the number of otic vesicle diameters between the optic cup and the otic vesicle itself. Because of the continuous growth of the optic cup and otic vesicles during head‐straightening, the value of ovl decreases progressively (Figure 15). For this reason, we defined four stages as follows: 4‐ovl, 3‐ovl, 2‐ovl, and 1‐ovl.

##### STAGE 17: 4‐ovl, 80 hpf (3.3 dpf)

**FIGURE 16:**
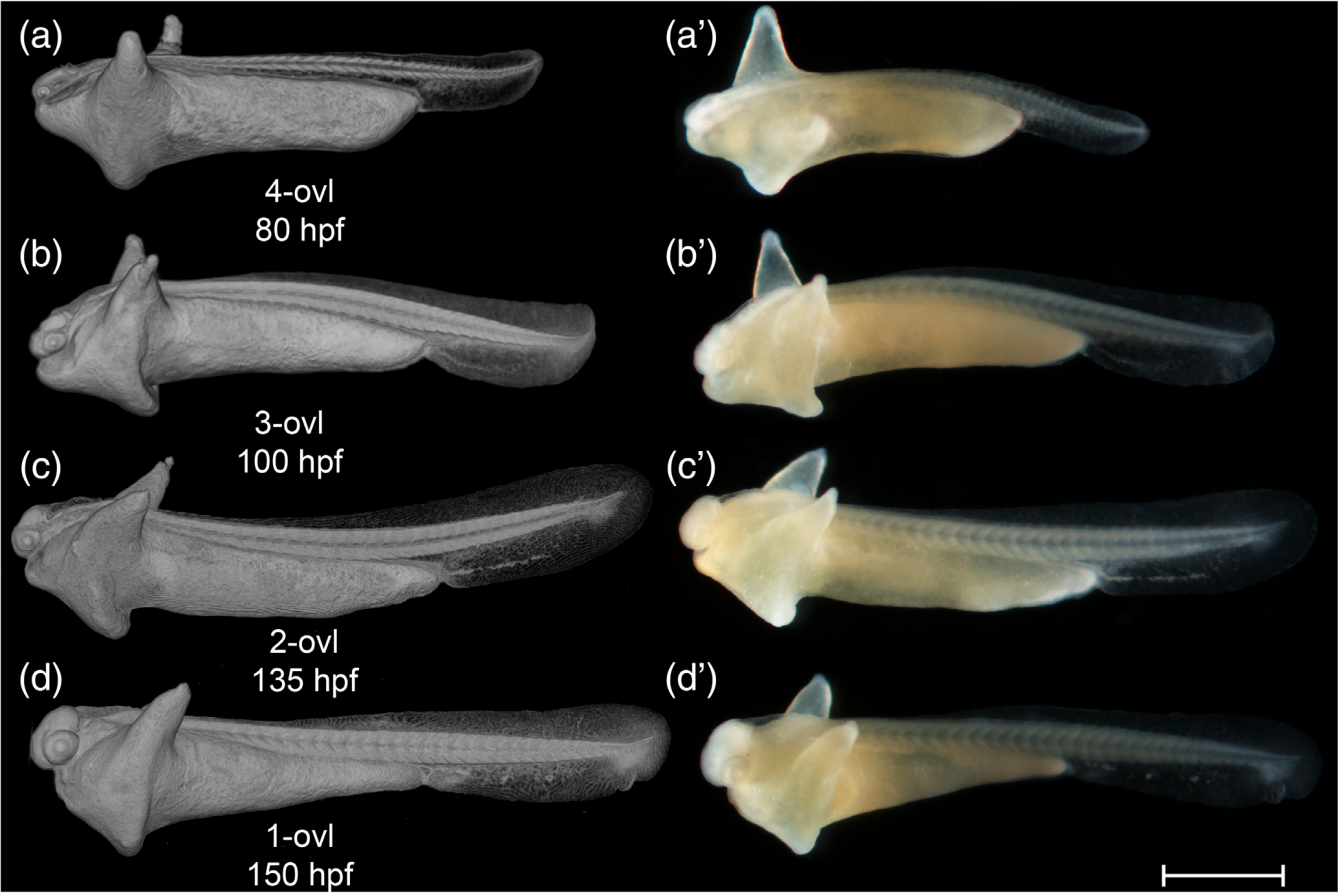
*Rhodeus ocellatus*, stages during the pharyngula period. (a–d) MicroCT images, volume rendering. (a'–d') Photomicrographs of the same embryo. (a and a') Stage 4‐ovl, 80 hpf. (b and b') Stage 3‐ovl, 100 hpf. (c and c') Stage 2‐ovl, 135 hpf. (d and d') Stage 1‐ovl, 150 hpf. Lateral view, dorsal up, head to the left. The photomicrographs are slightly rotated to the left along the primary axis in order to show both yolk sac extensions. Abbreviations: hpf, hours post‐fertilization; ovl, otic vesicle length. Scale bar = 1 mm

**FIGURE 17:**
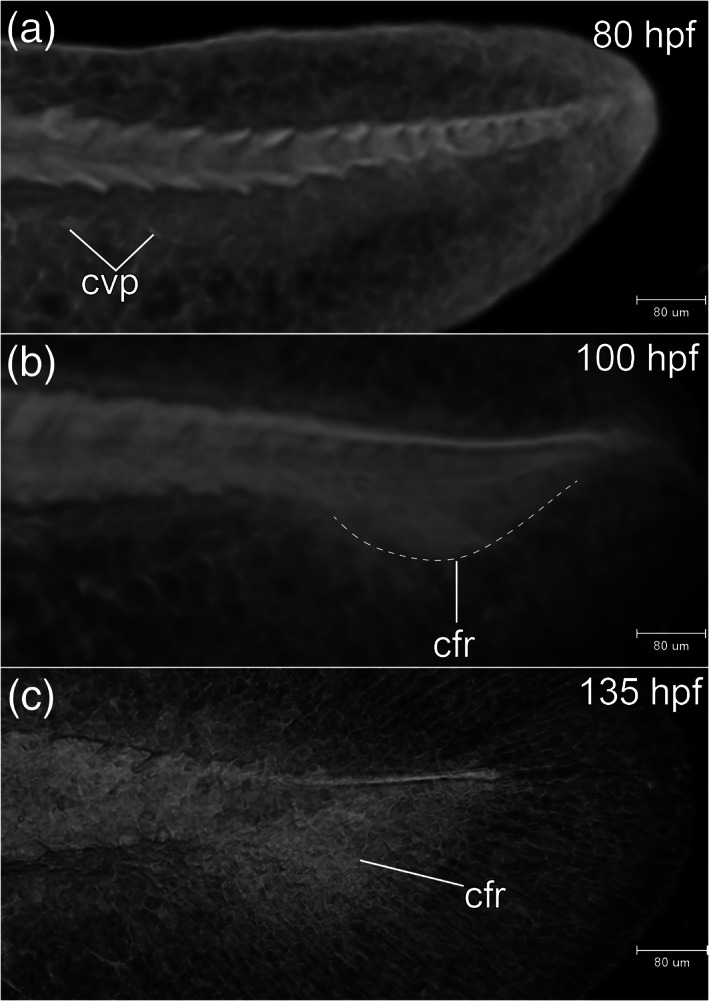
*Rhodeus ocellatus*, tail development, microCT images, virtual sections. (a–c) Lateral view, dorsal to the top, rostral left. (a) 4‐ovl, 80 hpf; (b) 3‐ovl, 100 hpf; (c) 2‐ovl, 135 hpf. Dashed line in (b) indicates the margin of the primordia of caudal fin rays. Abbreviations: cfr, primordia of caudal fin rays; cvp, caudal vein plexus

**FIGURE 18:**
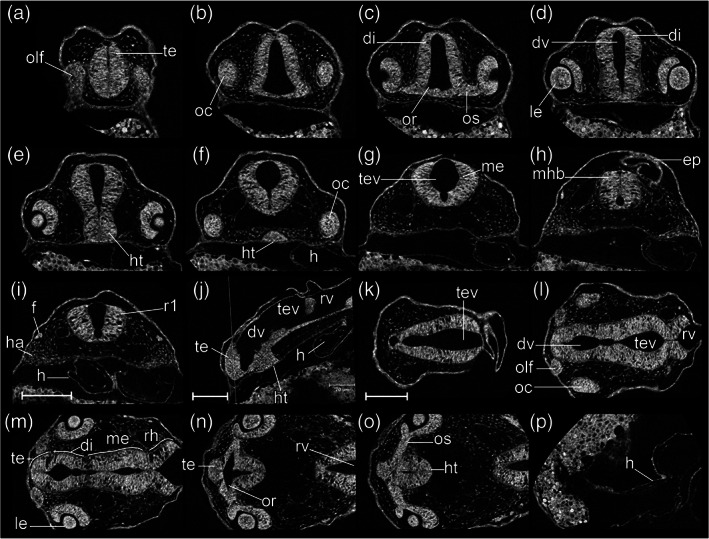
*Rhodeus ocellatus*, stages 4‐ovl, microCT images, virtual sections. (a–i) Transverse section views, dorsal towards the top, section plane indicated in (j). (j) Mid‐sagittal section view, rostral to the left, dorsal towards the top. (k–p) Coronal section views, from dorsal to ventral, rostral to the left. Abbreviations: di, diencephalon; dv, diencephalic ventricle; ep, epidermis; f, facial ganglion; h, heart; ha, hyoid arch cartilage; ht, hypothalamus; le, lens; me, mesencephalon; mhb, midbrain–hindbrain boundary; oc, optic cup; olf, olfactory placode; or, optic recess; os, optic stalk; r1 to 7, rhombomeres 1 to 7; rh, rhombencephalon; rv, rhombencephalic ventricle; te, telencephalon; tev, tectal ventricle; tv, telencephalic ventricle. Scale bars, 100 μm

All somites or myotomes are chevron‐shaped (Figure 16a). The caudal vein plexus appears in the anterior‐ventral region of the tail, just posterior to the caudal yolk extension (Figure 17a). The cloacal primordium is a funnel‐shaped structure near the base of the ventral yolk extension (Figure 16a). Rhombomere 7 is now distinct (Figure 15a). The trigeminal (V) ganglion is visible at the axial level of r2, and appears to be connected with the first (mandibular) pharyngeal arch. The pharyngeal arches are visible as three discrete cell condensations at this stage. The first is the mandibular arch, the second is the hyoid arch, and the third is the combined pharyngeal arches 1–5 (Figure 15a). The lens is completely detached from the overlying epidermis (Figure 18d). The tubular heart comprises an inner, endocardial layer, and an outer, trabecular, myocardial layer (Figure 18i). The heart tube migrates leftward and begins to loop dextrally (Figure 18p). In the Petri dish, the embryo can swim away a short distance if stimulated by a jet of water from a pipette. This stage is comparable to a stage intermediate between Kimmel stages *26‐somite* and *prim‐6*.

##### STAGE 18: 3‐ovl, 100 hpf (4.2 dpf)

**FIGURE 19:**
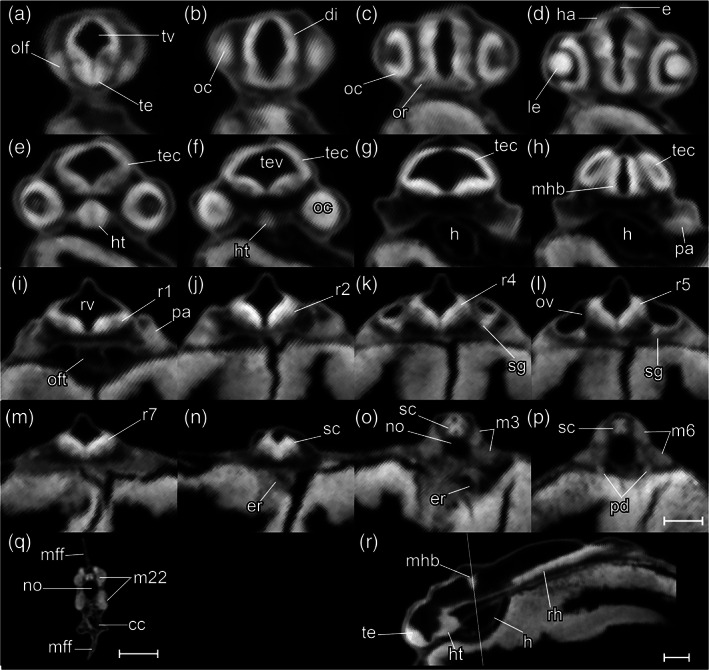
*Rhodeus ocellatus*, stage 3‐ovl, microCT images, virtual sections. (a–q) Transverse section views, dorsal towards the top, sections from rostral to caudal, direction of section plane indicated by the line in (r). (r) Mid‐sagittal section view, rostral to the left, dorsal towards the top. Abbreviations: cc, cloaca; di, diencephalon; e, epiphysis; er, endodermal rod; h, heart; ha, habenula; ht, hypothalamus; le, lens; m, myotome; mff, median fin fold; mhb, midbrain–hindbrain boundary; no, notochord; oc, optic cup; oft, outflow tract; olf, olfactory placode; or, optic recess; ov, otic vesicle; pa, pharyngeal arch; pd, pronephric duct; r1 to 7, rhombomeres 1 to 7; rh, rhombencephalon; rv, rhombencephalic ventricle; sc, spinal cord; sg, statoacoustic ganglion; te, telencephalon; tec, optic tectum; tev, tectal ventricle; tv, telencephalic ventricle. Scale bars, 100 μm

The tips of the wing‐like YSEs are directed caudally, and covered by skin warts (Figure 16b). The caudal yolk extension is markedly tapered at its caudal end (Figure 16b). The median fin fold in the tail becomes taller than the caudal yolk extension (Figure 16b). At the ventral base of the caudal fin fold, the prospective caudal fin rays are appearing (Figure 17b). The median fin fold at dorsal extends rostrally, its anterior‐rostral margin approaching the axial level of myotomes 6–8 (Figure 19p).

In the bitterling, the telencephalic ventricle undergoes eversion, as it does in other ray‐finned fishes including the zebrafish (Mueller & Wullimann, 2009; Wullimann & Puelles, 1999). This is in contrast to the evagination of the ventricle seen in amniotes (Folgueira et al., 2012). Therefore, instead of two lateral ventricles uniting in the midline, the bitterling has a large, fan‐shaped telencephalic ventricle everted dorsoventrally (Figures 15b and 19a). The isthmic constriction (mhb) is much deeper than in previous stages (compare Figure 15a,b). The olfactory placodes are oval, with their long axes parallel to the rostrocaudal axis (Figure 15b). The optic tectum expands to the lateral side, overlying the optic cups (Figure 15b).

A pair of common cardinal veins (ducts of Cuvier) is present, and contains blood flowing from the yolk sac to the inflow tract (sinus venosus). The heart has a regular heart beat and blood circulation. A solid endodermal rod, the primordium of the gut, is appearing (Figure 19n). The posterior lateral line primordia extend to the level of myotomes 5–6. This stage corresponds to Kimmel stage *prim‐5*.

##### STAGE 19: 2‐ovl, 135 hpf (5 dpf)

**FIGURE 20:**
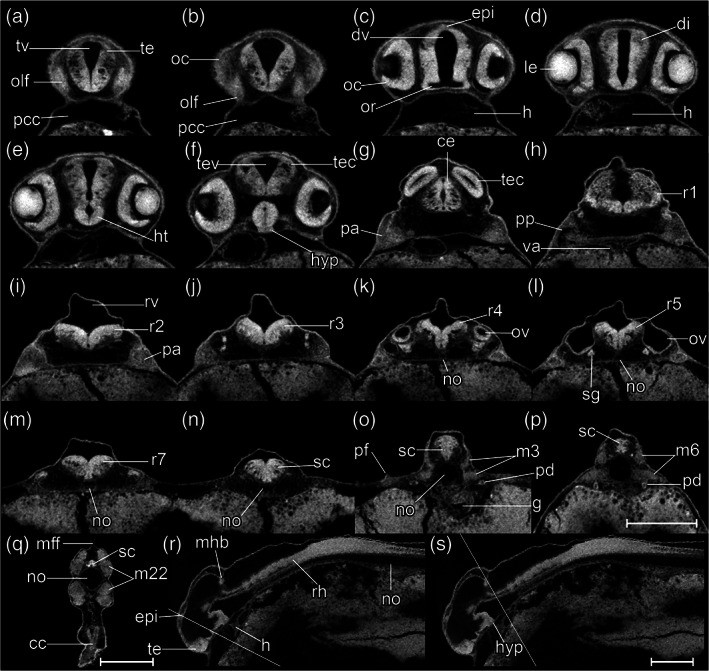
*Rhodeus ocellatus*, stage 2‐ovl, microCT images, virtual sections. (a–q) Transverse section views, dorsal towards the top, sections from rostral to caudal, direction of section plane from (a) to (f) indicated in (r); section plane in (g)–(q) indicated in (s); (r and s) mid‐sagittal section views, rostral to the left, dorsal towards the top. Abbreviations: cc, cloaca; ce, cerebellum; di, diencephalon; dv, diencephalic ventricle; epi, epiphysis; g, gut; h, heart; ht, hypothalamus; hyp, hypophysis; le, lens; lv, lateral ventricle; m, myotome; mff, median fin fold; mhb, midbrain–hindbrain boundary; no, notochord; oc, optic cup; olf, olfactory placode; or, optic recess; ov, otic vesicle; pa, pharyngeal arch; pcc, pericardial cavity; pd, pronephric duct; pf, pectoral fin bud; pp, pharyngeal pouch; r1 to 7, rhombomere 1 to 7; rh, rhombencephalon; rv, rhombencephalic ventricle; sc, spinal cord; sg, statoacoustic ganglion; te, telencephalon; tec, optic tectum; tev, tectal ventricle; va, ventral aorta. Scale bars, 200 μm

The pericardial cavity bulges prominently from the surface of the yolk sac (Figure 20a). In live specimens the common cardinal vein is red and contains flowing blood (Figure 16c'). Gill rudiments appear as shallow furrows rostral to the otic vesicle (Figure 16c). The cerebellum is clearly distinguishable at the axial level of r1 (Figures 15c and 20g).

In sagittal microCT virtual sections, the epiphysis appears as a swelling in the midline of the diencephalic roof plate (Figure 20r). The hypophysis is a small, well‐defined swelling descending in the ventral midline of the floor of the diencephalon (Figure 20f,r). Otic epithelium cells are condensing to form the sensory maculae (Figure 20k).

The outflow tract of the heart tube migrates to the axial level of the rostral margin of the optic cup (Figure 20a–h). The ventral aorta and the first pair of aortic arch arteries are recognizable in transverse sections (Figure 20h). The pectoral fin buds appear as cell patches at the level of myotome 3 at the base of the YSEs. These buds are indistinct in surface view but distinct in sections as condensed protrusions of the lateral plate mesoderm (Figure 20o). A lumen appears in the gut (Figure 20o). The migrating margin of the posterior lateral line primordia approaches myotome 10. This stage corresponds to Kimmel stage *prim‐10*.

##### STAGE 20: 1‐ovl, 150 hpf (6.25 dpf)

This stage represents the maximal extent of the YSEs before they regress at later stages (Figure 16d). The tail is now half the length of the body (Figure 16d). Blood vessels appear in the dorsal part of the tail (Figure 16d). At the ventral base of the tail, the primordia of the caudal fin rays are in a fan‐shaped array (Figure 16d). Sporadic melanocytes with faint pigment are appearing in the retina (Figure 16d').

**FIGURE 21:**
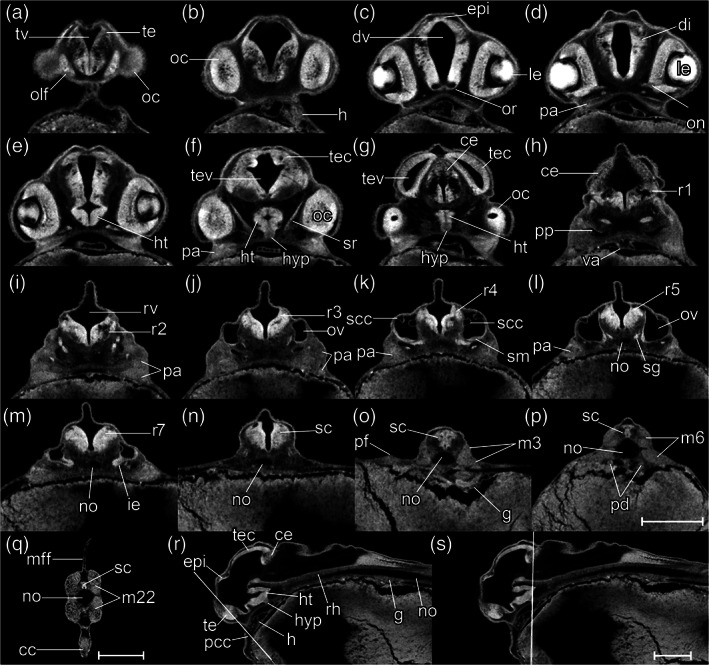
*Rhodeus ocellatus*, stage 1‐ovl, microCT images, virtual sections. (a)–(q) are transverse section view, dorsal towards the top, sections go from rostral to caudal, direction of section plane from (a) to (f) indicated in (r), section plane from (g) to (q) indicated in (s); (r) and (s) are mid‐sagittal section view, rostral to the left, dorsal towards the top. Abbreviations: cc, cloaca; ce, cerebellum; di, diencephalon; dv, diencephalic ventricle; epi, epiphysis; g, gut; h, heart; ht, hypothalamus; hyp, hypophysis; ie, pars inferior of inner ear; le, lens; no, notochord; oc, optic cup; olf, olfactory placode; or, optic recess; ov, otic vesicle; pa, pharyngeal arch; pcc, pericardial cavity; pf, pectoral fin bud; pp, pharyngeal pouch; r1 to 7, rhombomere 1 to 7; rh, rhombencephalon; rv, rhombencephalic ventricle; sc, spinal cord; scc, semicircular canal; sg, statoacoustic ganglion; sm, sensory maculae; te, telencephalon; tec, optic tectum; tev, tectal ventricle; tv: Telencephalic ventricle; va, ventral aorta; pd, pronephric duct; m, myotome; mff, median fin fold. Scale bars, 200 μm

The olfactory placode is semicircular (Figure 21a). In the otic vesicle, there are epithelial projections into the lumen from each wall forming the pillars of the semicircular canals (Figure 21k). The pars inferior of the developing inner ear (primordium of the lagena and saccule) are forming as a ridge on the ventromedial part of the otic vesicle (Figure 21m). The caudal end of the hypothalamus extends dorsally, towards the ventral surface of the medulla oblongata (Figure 21r). This displacement represents the maximum extent of the cephalic flexure. The pectoral fin bud is a shallow dome (Figure 21o). The anterior margin of the migrating posterior lateral line primordia approach myotome 24. This stage corresponds to Kimmel stage *prim‐25*.

#### 3.2.3 Organogenetic period

**FIGURE 22:**
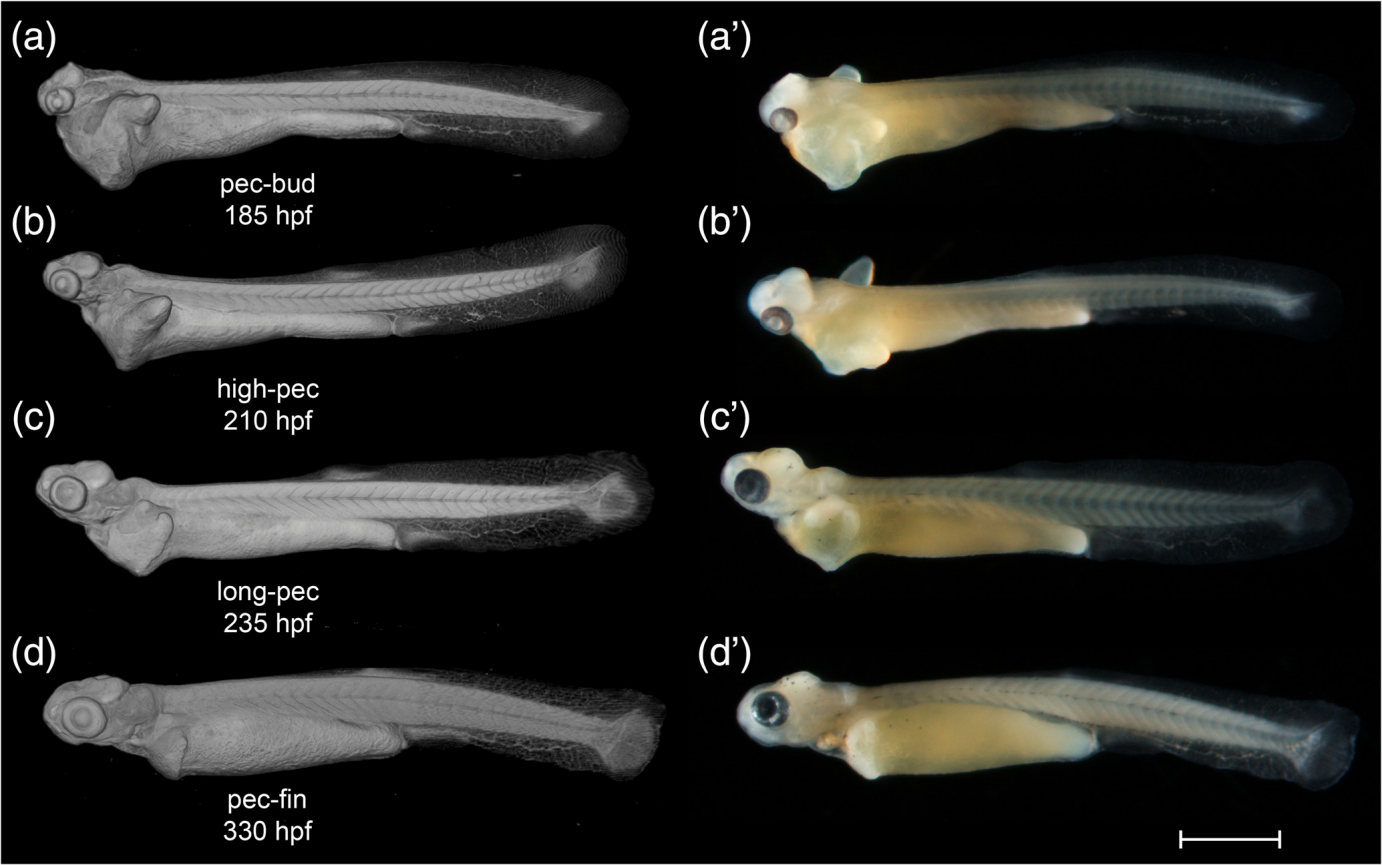
*Rhodeus ocellatus*, stages during the organogenetic period. (a)–(d) MicroCT images, volume rendering. (a') to (d') Photomicrographs of the same embryo. (a and a') Stage pec‐bud, 185 hpf. (b and b') Stage high‐pec, 210 hpf. (c and c') Stage long‐pec, 235 hpf. (d and d') Stage pec‐fin, 330 hpf. Lateral view, dorsal up, head to the left. Scale bar = 1 mm

**FIGURE 23:**
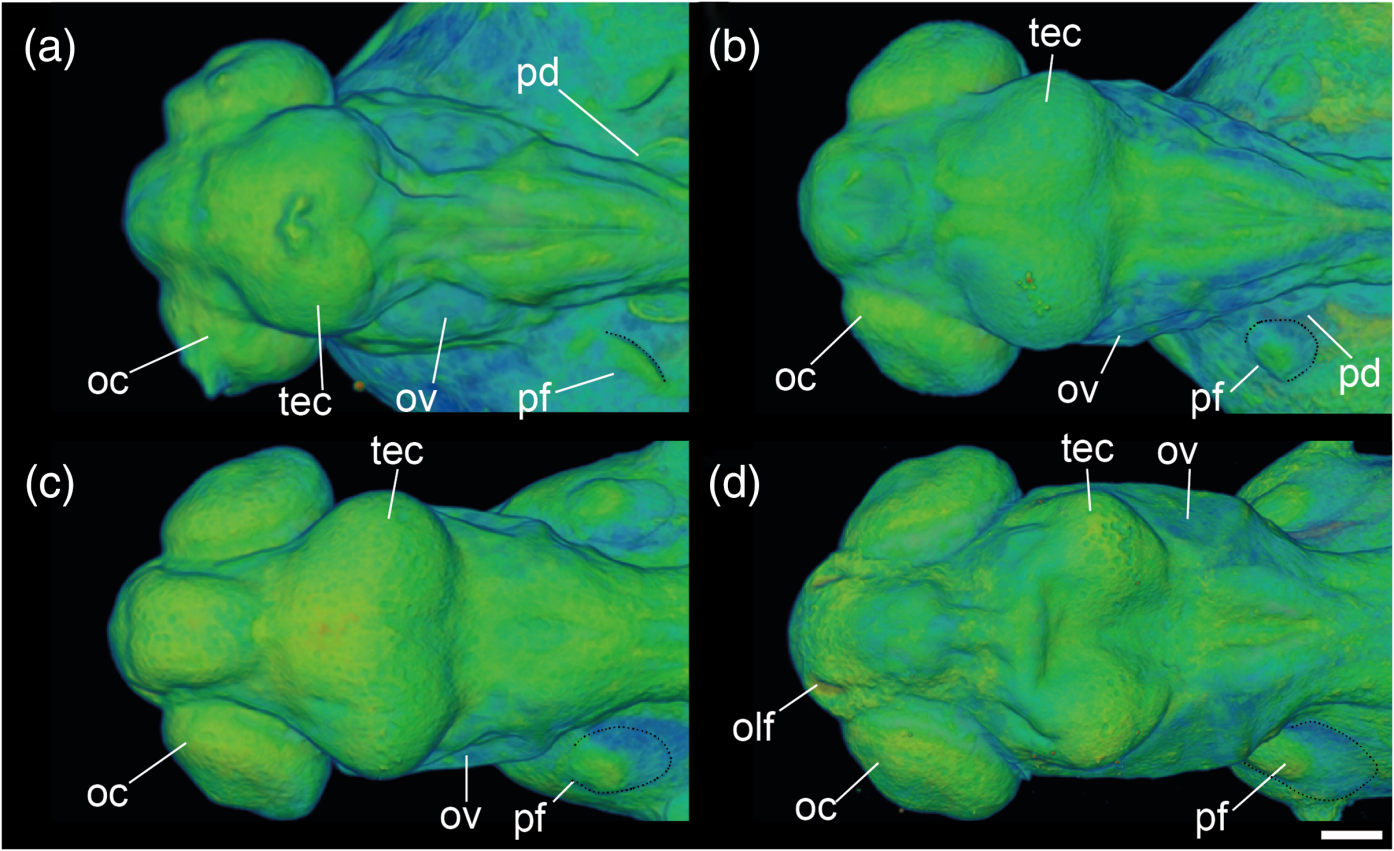
*Rhodeus ocellatus*, embryos during the organogenetic period, microCT images, pseudo‐colored volume‐renderings. Dorsal view, rostral to the left. Margins of left pectoral fin bud/fold are illustrated with a dotted line. (a) pec‐bud, 185 hpf; (b) high‐pec, 210 hpf; (c) long‐pec 235 hpf; (d) pec‐fin, 330 hpf. Abbreviations: le, lens; oc, optic cup; olf, olfactory pit; ov, otic vesicle; pd, pronephric duct; pf, pectoral fin bud/fold; tec, optic tectum. Scale‐bars, 100 μm in (a) and (b), 120 μm in (c) and (d)

Throughout the development of the previous (pharyngula) period, the body plan of the embryo was established. In the current period, regional development of organs is marked. We therefore define this as the “organogenetic” period. The key staging character for each stage is the morphology of the pectoral fin bud (Figures 22 and 23), and we have made this consistent with the Kimmel zebrafish stages to facilitate comparison.

**FIGURE 24:**
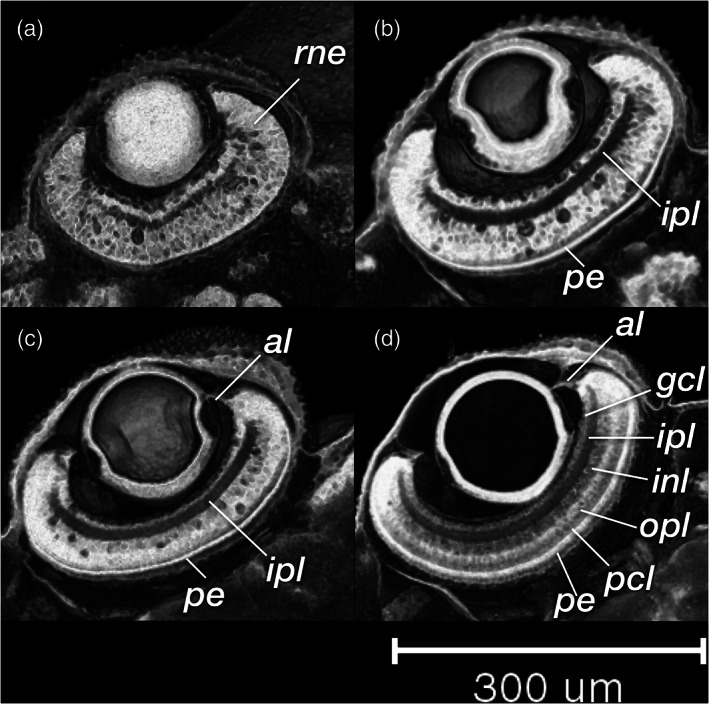
*Rhodeus ocellatus*, retinal lamination at different stages of development. Dorsal view, coronal section plane of volume rendering visualization. (a) 165 hpf; (b) 210 hpf; (c) 235 hpf; (d) 330 hpf. Abbreviations: al, annular ligament; gcl, ganglion cell layer; inl, inner nuclear layer; ipl, inner plexiform layer; le, lens; opl, outer plexiform layer; pcl, photoreceptor layer; pe, pigmented epithelium; rne, retina neuroepithelium

**FIGURE 25:**
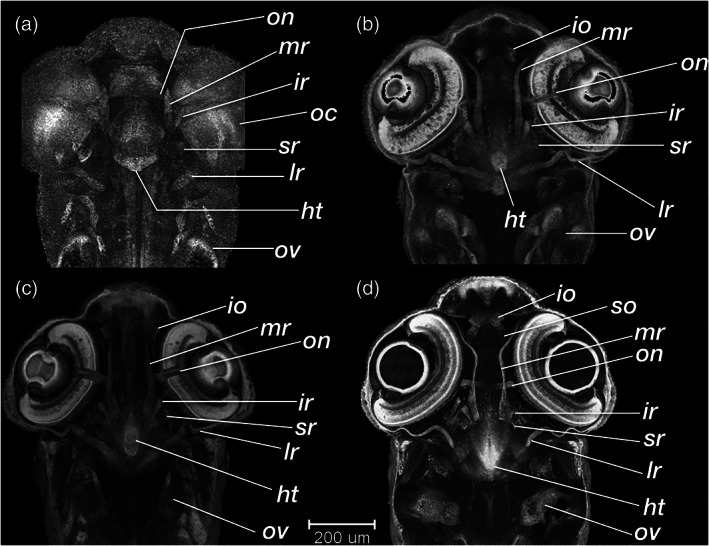
*Rhodeus ocellatus*, development of the extraocular muscles. (a) 165 hpf; (b) 210 hpf; (c) 235 hpf; (d) 330 hpf. Ventral views of coronal section planes at the level of the hypothalamus. Abbreviations: ht, hypothalamus; io, inferior oblique; ir, inferior rectus.; lr, lateral rectus; mr, medial rectus; oc, optic cup; on, optic nerve; ov, otic vesicle; so, superior oblique; sr, superior rectus

**FIGURE 26:**
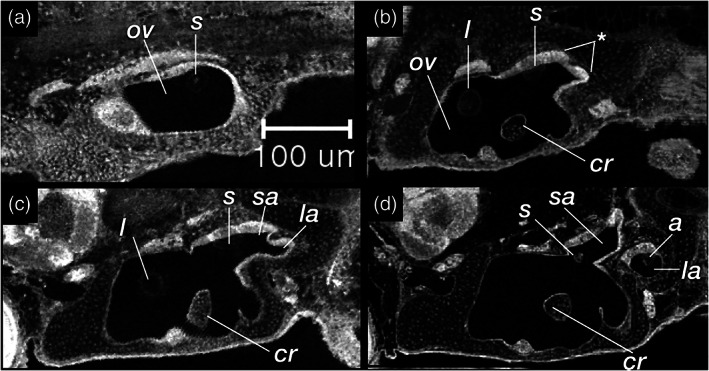
*Rhodeus ocellatus*, development of the pars inferior of the inner ear. Coronal sections of the ventral part of the ear. (a) 165 hpf. The anterior otolith lapillus and the posterior otolith sagitta are well formed. (b) 210 hpf. (c) 235 hpf, the protrusion dividing the lumen into two (d) 330 hpf. The asteriscus forms last in the chamber of lagena, and the saccule is separate from the utricle. Abbreviations: a, asteriscus; cr, common crus; l, lapillus; la, lagena; ov, otic vesicle; s, sagitta; sa, saccule; u, utricle; *, protrusion of saccule and lagena from the caudal margin of the otic vesicle wall

**FIGURE 27:**
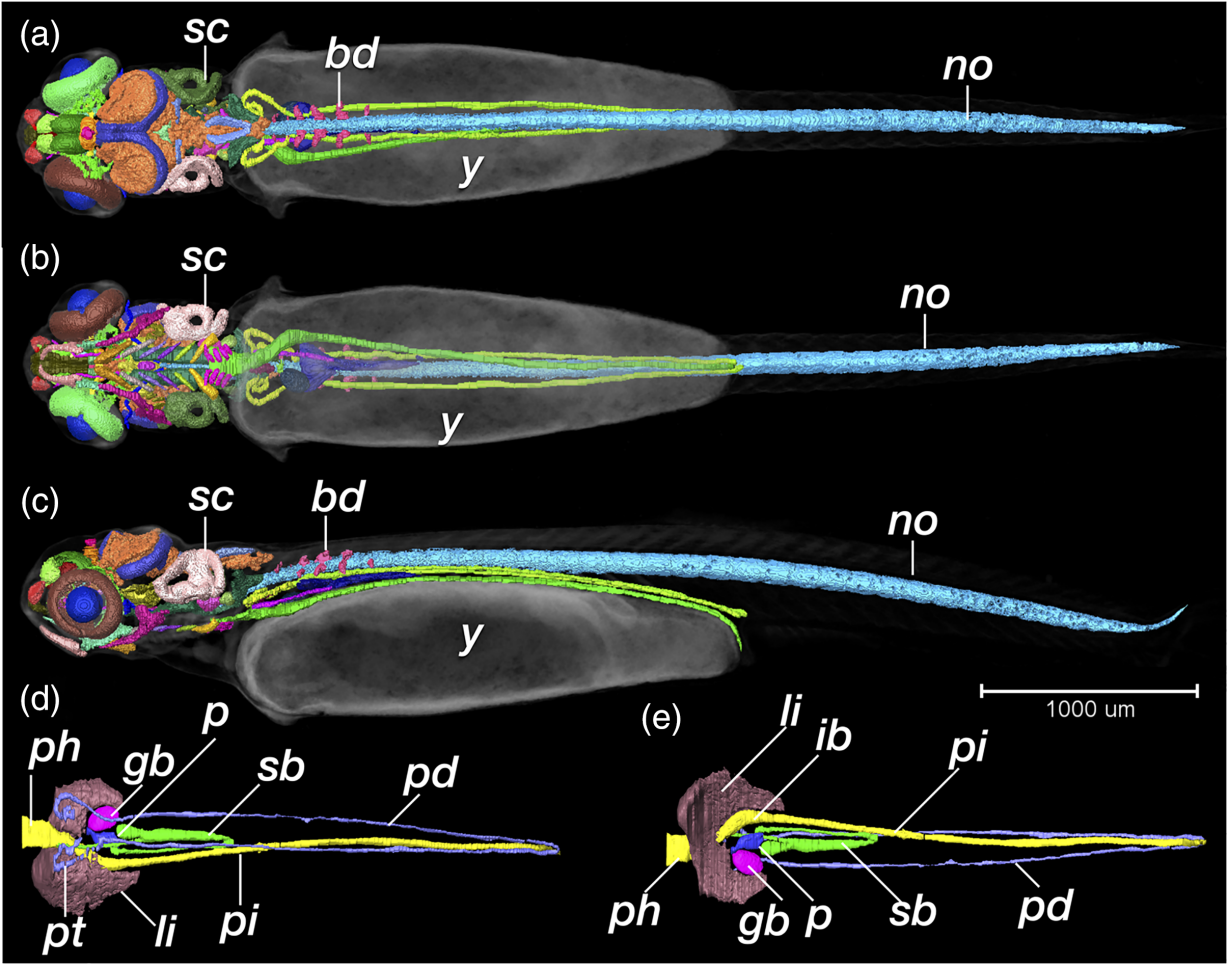
*Rhodeus ocellatus*, pec‐fin stage. (a and d) Dorsal views. (b and e) Ventral views. (c) Lateral view, rostral to the left. (a)–(c) are the overviews of segmentation result visualized by surface view, background tissue (including yolk) visualized by volume rendering (greyscale color‐map, transparent). (d and e) Segmented surface view of the digestive system the previously solid endodermal rod develops into an alimentary canal and gives rise to liver, gall bladder and pancreas buds, as well as the endodermal lining of the swim bladder. Abbreviations: bd, basidorsal cartilage; gb, gall bladder; ib, intestine bulb; li, liver; no, notochord; p, pancreas; pd, pronephric duct; ph, pharynx; pi, posterior intestine; pt, pronephric tubule; sb, swim bladder; sc, semicircular canal; y, yolk. Scale bar, 1 mm

**FIGURE 28:**
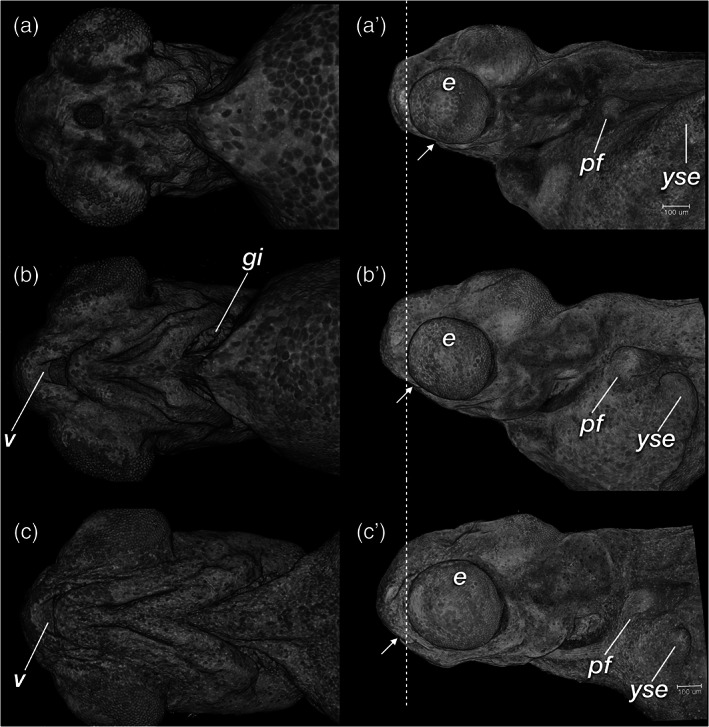
*Rhodeus ocellatus*, mouth protrusion and jaw extension during the organogenetic period. (a and a') 210 hpf the mouth opening located ventrally, approaching the rostral margin of the eye. (b and b') 235 hpf the jaw extends rostrally, now at to the rostral margin of eye cups. (c and c') 330 hpf. The mouth opening has now extended further rostral, beyond the rostral margin of the eye. (a and c) Ventral views; (a', c') left lateral views. Abbreviations: e, eye; gi, gill filaments; pf, pectoral fin bud; v, upper oral valve; yse, yolk sac extension. Dashed line, rostral margin of eye; arrows, mouth opening

During the organogenetic period, we observe lamination of the retina (Figure 24), formation of the extraocular muscles (Figure 25), differentiation of the pharyngeal cartilages (Meckel's cartilage, palatoquadrate, ceretohyal, hyosymplectic, and ceratobranchials, see Figure S1), morphogenesis of the semicircular canals, and formation of the inferior part of the inner ear (Figure 26). The gut was a solid endodermal rod in the pharyngula period, but in the organogenetic period it develops into an alimentary canal with liver, gall bladder and pancreatic primordia (Figure 27). The swim bladder also develops (Figure 27). At the end of this period, as the bitterling approaches the end of its parasitic life. It has a gaping mouth (Figure 28), a mobile lower jaw, gill filaments on the gill arches, and pointed pharyngeal teeth. The yolk mass is depleted and the wing‐like YSEs regresses completely (Figure 22).

##### STAGE 21: Pec‐bud, 185 hpf (7.7 dpf)

**FIGURE 29:**
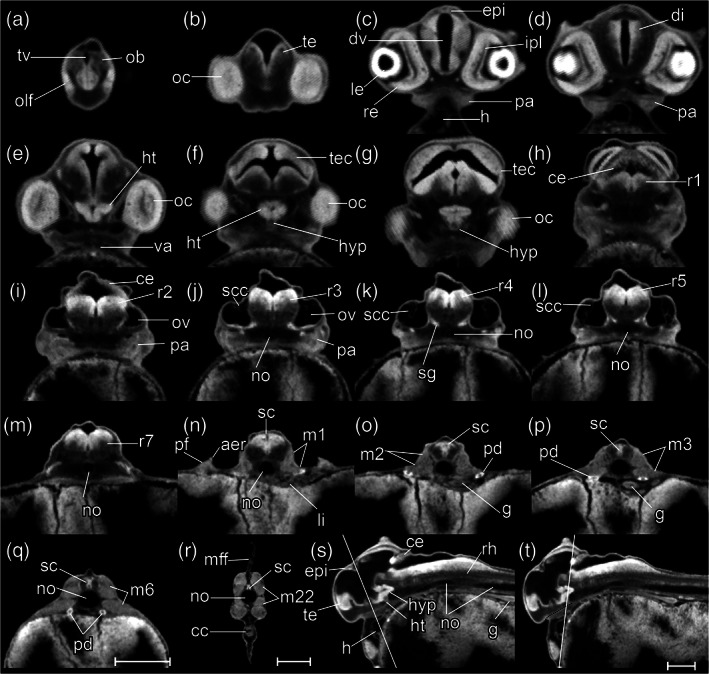
*Rhodeus ocellatus*, stage pec‐bud, microCT images, virtual sections. (a)–(r) are transverse section view, dorsal towards the top, sections go from rostral to caudal, direction of section plane from (a) to (f) indicated in (s), section plane from (g) to (r) indicated in (t); (s) and (t) are mid‐sagittal section view, rostral to the left, dorsal towards the top. Abbreviations: aer, apical ectodermal ridge; cc, cloaca; ce, cerebellum; di, diencephalon; epi, epiphysis; g, gut; h, heart; ht, hypothalamus; hyp, hypophysis; ipl, inner plexiform layer; li, liver; m, myotome; mff, median fin fold; no, notochord; Ob, olfactory bulb; oc, optic cup; le, lens; olf, olfactory epithelium; ov, otic vesicle; pa, pharyngeal arch; pd, pronephric duct; pf, pectoral fin bud; r1 to 7, rhombomere 1 to 7; re, retina; rh, rhombencephalon; sc, spinal cord; scc, semicircular canal; sg, statoacoustic ganglion; te, telencephalon; tec, optic tectum; tv, telencephalic ventricle. Scale‐bars equal to 200 μm

In dorsal view, the pectoral fin bud is dome‐shaped (Figure 23a). The height of the pectoral fin bud is equal to its dorsoventral width (Figures 23a and 29n). The apical ectodermal ridge is discernible (Figure 29n). Based on the morphology of the pectoral fin bud, we name this stage “pec‐bud.” The YSEs taper ventro‐dorsally. They still extend dorsally but not beyond the level of the dorsal margin of the eye (Figure 22a). Two cell condensations are recognizable in the median fin fold; these are the primordia of the dorsal and anal fins (Figure 22a). On the yolk sac, the bilateral common cardinal vein is fan‐shaped, and in live specimens contains vigorously flowing blood. Retinal pigment is now distinct (compare Figures 22a' and 16d').

The olfactory bulb is forming (Figure 29a). What we presume to be the inner plexiform layer of the retina is distinct (Figure 29c,d). There is no mouth opening; the mouth is indicated by a shallow groove (Figure 29s). The protrusions forming anterior and posterior semicircular canals are fused; the common crus and lateral semicircular canal are developing (Figure 29j–l). The liver is visible (Figure 29n). This stage is intermediate between Kimmel *prim‐25* and *high‐pec* stages.

##### STAGE 22: High‐pec, 210 hpf (8.75 dpf)

**FIGURE 30:**
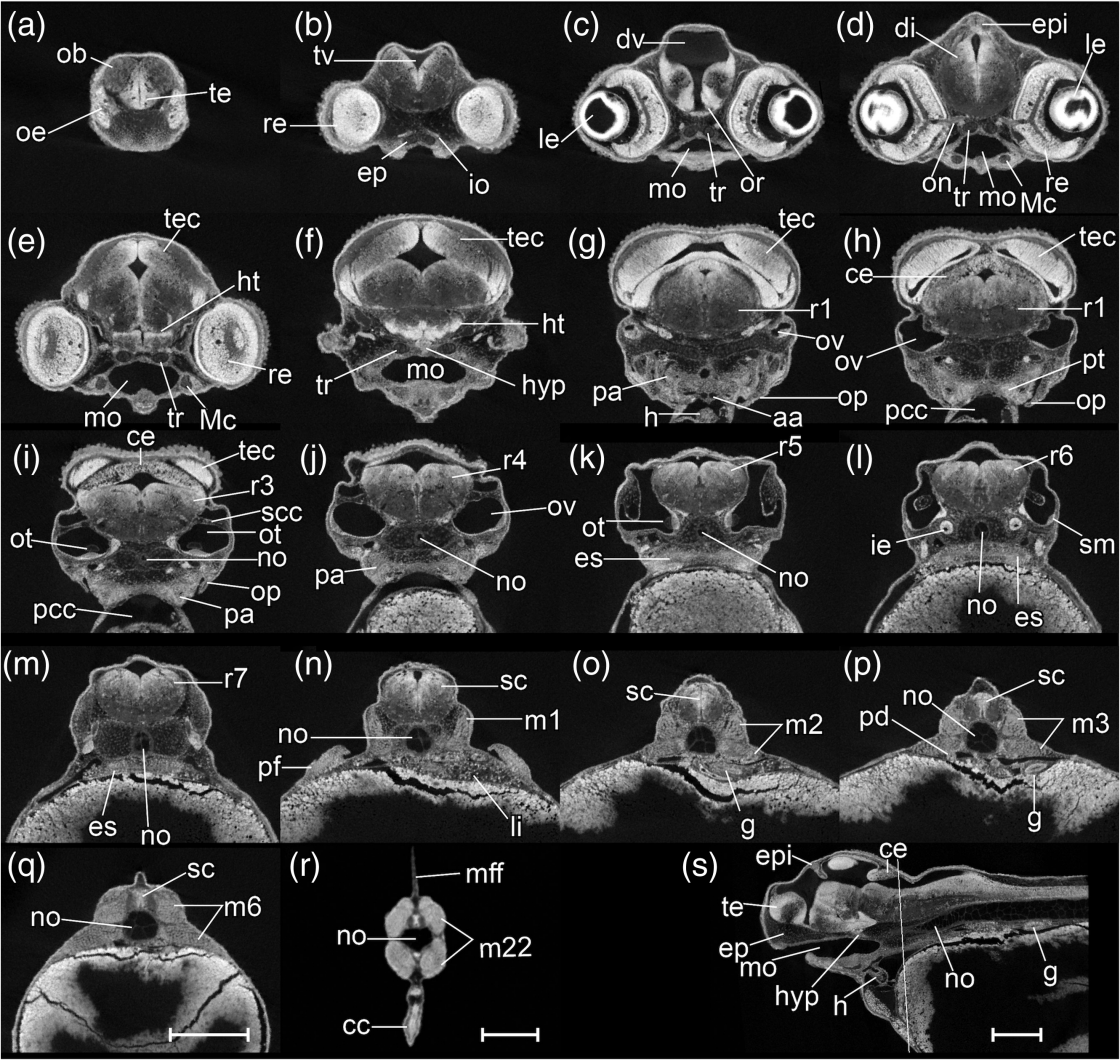
*Rhodeus ocellatus*, stage high‐pec, microCT images, virtual sections. (a–r) Transverse section views, dorsal towards the top, sections from rostral to caudal, direction of section plane indicated in (s). (s) Mid‐sagittal section view, rostral to the left, dorsal towards the top. Abbreviations: aa, arch artery; cc, cloaca; ce, cerebellum; di, diencephalon; ep, ethmoid plate; epi, epiphysis; es, esophagus; g, gut; h, heart; ht, hypothalamus; hyp, hypophysis; ie, pars inferior of inner ear; io, inferior oblique muscle; le, lens; li, liver; m, myotome; Mc, Meckel's cartilage; mff, median fin fold; mo, mouth; no, notochord; ob, olfactory bulb; oe, olfactory epithelium; on, optic nerve; op, operculum; or, optic recess; ot, otolith; ov, otic vesicle; pa, pharyngeal arch; pcc, pericardial cavity; pd, pronephric duct; pf, pectoral fin bud; pt, pharyngeal teeth; r1 to 7, rhombomere 1 to 7; re, retina; rh, rhombencephalon; sc, spinal cord; scc, semicircular canal; sg, statoacoustic ganglion; sm, sensory maculae; te, telencephalon; tec, optic tectum; tr, trabeculae cranii; tv, telencephalic ventricle. Scale bars, 200 μm

**FIGURE 31:**
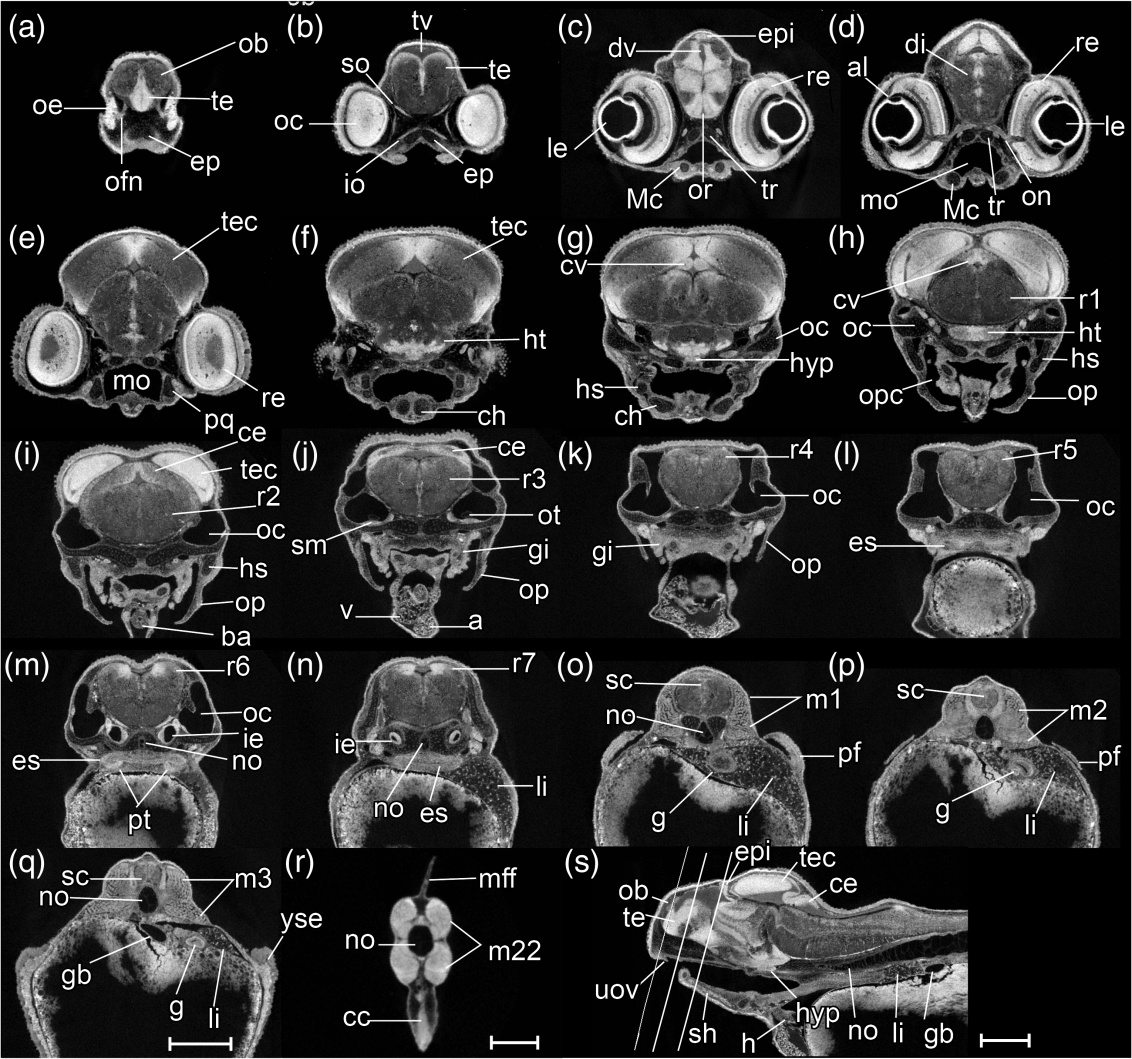
*Rhodeus ocellatus*, stage long‐pec, microCT images, virtual sections. (a–r) Transverse section views, dorsal towards the top, sections go from rostral to caudal, direction of section plane indicated in (s). (s) Mid‐sagittal section view, rostral to the left, dorsal towards the top. Abbreviations: a, atrium; al, annular ligament; ba, bulbus arteriosus; cc, cloaca; ce, cerebellum; ch, ceratohyal cartilage; di, diencephalon; ep, ethmoid plate; epi, epiphysis; es, esophagus; g, gut; gb, gall bladder; gi, gill; h, heart; hs, hyosymplectic cartilage; ht, hypothalamus; hyp, hypophysis; ie, pars inferior of inner ear; io, inferior oblique muscle; le, lens; li, liver; m, myotome; Mc, Meckel's cartilage; mff, median fin fold; mo, mouth; no, notochord; ob, olfactory bulb; oc, otic capsule; oe, olfactory epithelium; ofn, olfactory nerve; on, optic nerve; op, operculum; opc, opercular cavity; or, optic recess; ot, otolith; pa, pharyngeal arch; pcc, pericardial cavity; pd, pronephric duct; pf, pectoral fin bud; pq, palatoquadrate arch cartilage; pt, pharyngeal teeth; r1 to 7, rhombomere 1 to 7; re, retina; sc, spinal cord; scc, semicircular canal; sg, statoacoustic ganglion; sh, sternohyoid muscle; sm, sensory maculae; so, superior oblique muscle; te, telencephalon; tec, optic tectum; tr, trabeculae cranii; tv, telencephalic ventricle; uov, upper oval valve; v, cardiac ventricle; yse, yolk sac extension. Scale bar, 200 μm

Pectoral fin bud: (i) mesenchymal condensations at the central of the fin bud (Figure 30n), (ii) height greater than width (Figure 23b). Dense retinal pigmentation, except in the region around the lens, giving the appearance, in light microscopy of whole embryos, of a black ring encircling the limpid lens (Figure 22b'). The pericardial cavity now lies rostral to the yolk mass and so the heart is visible in lateral view (Figures 22b and 30s). Olfactory pit a shallow groove (Figures 22b and 30a). The mouth a small opening, not yet gaping (Figures 30c,d,s and 28a). No gill filaments present on the branchial arches (Figures 30g and 28a). Rudiments of pharyngeal teeth appearing on the 5th branchial arch (Figure 30h). Branchial clefts not yet open (compare Figures 30h and 31h). Cells in the liver have the histological features of hepatocytes (Figure 30n). A common chamber of the saccule and lagena appears (Figure 26b). This stage corresponds to Kimmel *high‐pec* stage, on the basis of the morphology of the pectoral fin bud.

##### STAGE 23: Long‐pec, 235 hpf (9.8 dpf)

The apical ectodermal ridge of the pectoral fin develops into the fin fold (Figures 23c and 31o). Chondrocytes are differentiating in the pectoral girdle (Figure 31o). The YSEs shrinks to vestigial bumps (Figures 22c and 31q). Melanophores are differentiating in a rostrocaudal gradient in the skin, and are most prominent on the dorsal surface of the head (Figure 22c'). The entire retinal pigment layer is pigmented (Figure 22c').

The olfactory epithelium of the bowl‐shaped olfactory pit is connected to the olfactory bulb by a distinct olfactory nerve (Figure 31a). The anterior portion of the trabeculae cranii expands and fuses with the ethmoid plate to form the trabecula communis (Figure 31a–c). The upper oral valve appears on the pharyngeal aspect of the upper jaw (Figure 31s). The jaw becomes extended rostrally, and the mouth opening therefore becomes located at the axial level of the rostral margin of the optic cup (Figure 28b,b'). The annular ligament appears in the angle between the cornea and the iris (Figures 31d and 24c). The opercular cavity expands and opens into the buccal cavity (Figure 31h). Buds of developing gill filaments are present on all gill arches (Figure 31j,k). The saccule and lagena are now distinct from each other (Figure 26c). A hollow gallbladder appears ventral to the intestine (Figure 31q). This stage corresponds to Kimmel *long‐pec* stage.

##### STAGE 24: Pec‐fin, 330 hpf (13.75 dpf)

**FIGURE 32:**
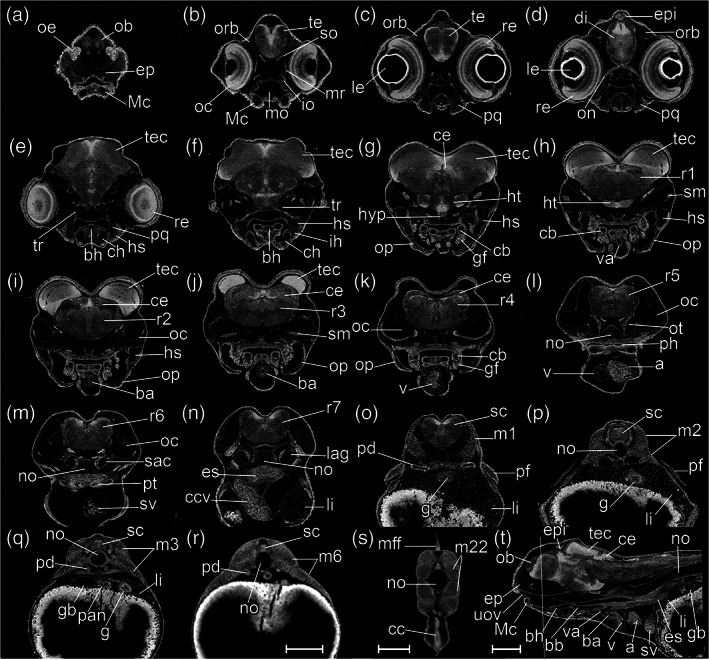
*Rhodeus ocellatus*, stage pec‐fin, microCT images, virtual sections. (a)–(s) are transverse section view, dorsal towards the top, sections go from rostral to caudal, direction of section plane indicated in (t); (t) is mid‐sagittal section view, rostral to the left, dorsal towards the top. Abbreviations: a, atrium; ba, bulbus arteriosus; bb, basibranchial cartilage; bh, basihyal cartilage; cb, ceratobranchial cartilages; cc, cloaca; ccv, common cardinal vein (duct of Cuvier); ce, cerebellum; ch, ceratohyal cartilage; di, diencephalon; ep, ethmoid plate; epi, epiphysis; es, esophagus; g, gut; gb, gall bladder; gf, gill filament; h, heart; hs, hyosymplectic cartilage; ht, hypothalamus; hyp, hypophysis; ih, interhyal cartilage; io, inferior oblique muscle; lag, lagena; le, lens; li, liver; m, myotome; Mc, Meckel's cartilage; mff, median fin fold; mo, mouth; mr, medial rectus muscle; no, notochord; ob, olfactory bulb; oc, otic capsule; oe, olfactory epithelium; ofn, olfactory nerve; on, optic nerve; op, operculum; opc, opercular cavity; or, optic recess; orb, orbital cartilage; ot, otolith; p, proctodeum; pan, pancreas; pcc, pericardial cavity; pd, pronephric duct; pf, pectoral fin bud; ph, pharynx; pq, palatoquadrate arch cartilage; pt, pharyngeal teeth; r1 to 7, rhombomere 1 to 7; re, retina; sac, saccule; sc, spinal cord; scc, semicircular canal; sg, statoacoustic ganglion; sm, sensory maculae; so, superior oblique muscle; sv, sinus venosus; te, telencephalon; tec, optic tectum; tr, trabeculae cranii; uov, upper oval valve; v, cardiac ventricle; va, ventral aorta. Scale bar, 200 μm

Pectoral apical fin folds have the form of asymmetric blades (Figure 23d). Iridophores are present in the iris giving the appearance of a reflective ring around the lens (Figure 22d'). On the trunk and tail, melanophores organize into a lateral stripe at the level of the horizontal myoseptal boundaries (Figure 22d'). Ventral melanocytes appear on the caudal end of the yolk sac (Figure 22d'). In concert with the extension of the jaw (Figure 28), the pericardial cavity extends further rostrally. The heart is elongated in its rostrocaudal axis; this is in contrast to the previous stage, in which the heart was oriented in the dorsoventral plane (compare Figures 31s and 32t). Therefore, live specimens (Figure 22d'), in lateral view (Figure 32t), show blood flow in the common cardinal vein (Figure 32n), sinus venosus (Figure 32m), atrium (Figure 32l), ventricle (Figure 32k), bulbus arteriosus (Figure 32i,j), ventral aorta (Figure 32h), and branchial arch arteries (Figure 32g).

In the chondrocranium, the orbital cartilage grows anteroventrally from the epiphyseal bar to join the edge of the ethmoid plate (Figure 32b–d). Although the orbital cartilage appeared in the previous stage, it is only in the current stage that the rostral connection to the ethmoid plate and caudal connection to the epiphyseal bar are distinct (data not shown). The medial basihyal and basibrancial appear in cartilage (Figure 32f,t) and the angle between the bilateral ceratohyal cartilage is acute (compare Figure S1a,c). The fifth branchial arch is distinct and carries four pairs of pharyngeal teeth (Figure S1). The asteriscus otoliths form in the chamber of the lagena (Figures 32n and 26d). The swim bladder is not yet inflated (Figure 27d,e). The first four pairs of basidorsal cartilages develop but are not differentiated as the Weberian apparatus (Figure 27). This stage corresponds to Kimmel stage *pec‐fin* stage, on the basis of fin morphology.

## 4 DISCUSSION

We have described 24 stages in the development of the rosy bitterling using microCT and have made the stages comparable to the Kimmel stages for the zebrafish. These corresponding developmental stage series laying the foundation for our subsequent research, a comparison of the sequence of developmental events with sequences in other non‐parasitism teleost based on parsimony analysis (Ito et al., 2019; Jeffery et al., 2005). Sequence heterochrony (changes in the order in which events occur) is an important mechanism for the evolution of development (Bininda‐Emonds et al., 2002; Mabee et al., 2000). Our study demonstrates the value of microCT in developmental biology. In addition to being relatively time‐efficient compared with routine histology, it is a non‐destructive technology. For species that were previously difficult to study because of limited material, microCT scans provide a wealth of morphological data and readily yield 3‐D information.

### 4.1 The body direction in relation to the polarity of the chorion

According to Suzuki (1958), the time‐window in which bitterling eggs can be fertilized is about 30 min after the egg has been activated when contacting water; sperms remain viable for only 7 min after contact with water. Egg activation is an irreversible process and is independent of the presence of sperm (Kunz, 2004). Once activated, the chorion becomes inflated and lifts from the egg surface (Figure 3). A funnel‐shaped micropyle, a specialization of the chorion, connects the chorion and egg surface, and serves as a passage for the sperms to fertilize the egg (Suzuki, 1961). When the female bitterling oviposits eggs inside the host mussel, these eggs are inevitably activated. Therefore, a successful fertilization requires that the male bitterling releases sperm near the host mussel within 30 min and the sperms find their way to the micropyle.

The chorion of the rosy bitterlings eggs is bulb‐shaped (Figures 3 and 4). The micropyle is always at the narrower stalk pole (Suzuki, 1961). As we observed, during the hatching period, the head always emerges from the chorion at its wider (bulb) side (Figure 4o). One explanation of the position of the micropyle could be that it facilitates fertilization. The eggs of bitterlings are deposited by the female using a long ovipositor; the eggs in each clutch are therefore arranged in a single row in the water tube of the mussel gill (Kunz, 2004). Kunz's hypothesis is: this polarity ensures that the micropyle of one egg faces the non‐micropyle end of the next to avoid sperm competition. We found out that the embryos always hatched out from the chorion from the opposite side of the micropyle, therefore the newly‐hatched embryos line in the water tube in the same fashion as the eggs, which thus reduces potential oxygen and space competition between embryos.

### 4.2 Notochord vacuolization is not necessary for body straightening

During the process of body elongation, especially from stages s‐18 to s‐32, the notochord elongates and expands in diameter because of vacuolization of the inner layer of notochordal cells. The notochord first has a typical “stack‐of‐coins” appearance at the s‐18 stage (Adams et al., 1990; Koehl et al., 2000), suggesting that the subsequent vacuolization of notochord plays an important role for body elongation by providing a mechanical force needed for straightening of the body axis along the RC axis (Stemple, 2005). Ellis et al. (2013) argued against this hypothesis, providing in vivo experimental evidence indicating that the notochord is not necessary for embryonic straightening.

Our results are consistent with the conclusions of Ellis et al. (2013) because the straightening of bitterling body axis takes place during the neurula extension and neurula migration periods, both of which are complete before the notochord becomes vacuolized. We acknowledge that a temporal correlation is not sufficient proof of the causal relationship of events. However, in this case, the temporal relationship is a dependent one and therefore argues against the mechanical hypothesis.

### 4.3 The yolk extension forms independently of body straightening

In the zebrafish, the straightening of the body axis (from its original conformation of being curved over the yolk sac) overlaps temporally with the formation of the yolk extensions (Virta, 2009). The trunk of the zebrafish straightens from the somite‐13 stage to the somite‐26 stage, and begins simultaneously with formation of the yolk extension (Kimmel et al., 1995; Virta, 2009; Virta & Cooper, 2009; Virta & Cooper, 2011). Virta (2009) conducted agarose immobilization of zebrafish embryos and found that the YE will form even when the trunk is prevented from straightening by the agarose.

In the bitterling, the morphogenetic movements that form the yolk extensions (YE) happen simultaneously with the straightening of the body axis. After that, extension of the YSEs dorsally occurs separately (in the somitogenesis period), without concomitant morphological changes in the body axis. For these reasons, we agree with Virta (2009) that YE formation can occur without concomitant straightening of the trunk.

### 4.4 Post‐displacement of eye development

Our study described the development of the eye in the rosy bitterling at the microanatomic level for the first time. Compared to the zebrafish (Easter Jr. & Nicola, 1996; Schmitt & Dowling, 1994, 1999), we find that the developmental timeline of early eye morphogenesis between the zebrafish and bitterling is broadly similar. The degree of ventral displacement of the optic primordium is similar between the bitterling at the 10‐somite stage (Figure 9c) and the zebrafish at the 11–12 somite stages. In the 16–17 somite stages of zebrafish, the lens placodes appear, and this event takes place at the corresponding 28‐somite stage of the bitterling (Figure 12d). However, in the zebrafish, the lens is completely detached from the surface ectoderm at the prim‐5 stage (24 hpf); this event occurred earlier in bitterling development at the 4‐ovl stage (Figure 18d).

It is worth noting that retinal pigmentation begins in the zebrafish at the prim‐5 stage. However, there is no sign of retinal pigmentation in the bitterling at the corresponding stage (Figure 16b'). The similar pigmentation level in the bitterling does not appear until 150 hpf (Figure 16d'), at a stage comparable to the prim‐25 stage of the zebrafish (36 hpf). Although it is obvious that, in the bitterling, the appearance of retinal pigmentation is post‐displaced compared with the development of the zebrafish, we are cautious not to draw the conclusion that the development of the eye in the bitterling is delayed. The development and maturity of the visual system, as shown in behavioral studies in the zebrafish, is based on visually evoked startle and optokinetic responses (Easter Jr. & Nicola, 1996). These responses not only require retinal pigmentation, but also retinal lamination (Figure 24), and formation of extraocular muscles (Figure 25) at later stages.

### 4.5 Pre‐displacement of hearing

Our study also analyzed the process of inner ear development in bitterling species at the microanatomic level for the first time. Comparing our results with ear development in zebrafish (Bever & Fekete, 2002; Haddon & Lewis, 1996; Riley & Moorman, 2000; Whitfield et al., 2002), we find that they take place at a comparable stage, although the morphogenesis of the pars inferior of the inner ear is strikingly pre‐displaced in bitterling development. Specifically, in the bitterling, the induction of the otic placode is at the 10‐somite stage (Figure 9d), the same as in the zebrafish (9–10 somite stages; (Haddon & Lewis, 1996; Whitfield et al., 2002). The cavitation of the otic vesicle becomes obvious at the 28‐somite stage (Figure 12g) in the bitterling and also at the correspondence stage in the zebrafish (18 somite stage). The neuroblast cells which will differentiate into the statoacoustic ganglion delaminate from the otocyst at the 35‐somite stage (Figure 14g) in the bitterling as well as the correspondence 26‐somite stage in the zebrafish (Haddon & Lewis, 1996; Whitfield et al., 2002).

In bitterling, the morphogenesis of the semicircular canals begins with the protrusion of the pillars at the 1‐ovl stage (Figure 21k) and then these protrusions fuse at the high‐pec stage (Figure 29l). The same process happens in the zebrafish from 42 to 72 hpf, slightly later than in the bitterling. More strikingly is the separation of the lagena from the sacculolagenar pouch; this takes place during the embryonic development of bitterling at the pec‐fin stage (330 hpf, 13.75 dpf), while in zebrafish, it takes place much later, during larval development (by 15 dpf; (Bever & Fekete, 2002; Whitfield et al., 2002).

The third otolith (asteriscus of the lagena), starts to forms at the pec‐fin stage in the bitterling, whereas the same event happens much later (9–17 dpf) in different studies of zebrafish depending on the strain (Bever & Fekete, 2002; Riley & Moorman, 2000; Whitfield et al., 2002). According to one study of otolith development and vestibular function in the zebrafish (Riley & Moorman, 2000), the utricular otolith is necessary and sufficient for vestibular function and survival in the zebrafish, whereas the sacculus and lagena otoliths function primarily in hearing. Therefore, we expect a pre‐displacement of hearing development in the bitterling, which may be related to brood parasitism life and development in a dark environment where hearing is more useful than vision. This in turn would also explain why visual development appears to be delayed in the bitterling.

### 4.6 MicroCT in developmental biology

The bitterling is not an easy species to study. Its YSEs and its large, opaque yolk mass are in contrast to the small, transparent early stages of zebrafish development. It is therefore much more difficult to observe with optical microscopy. We found that it is not feasible to manipulate differential interference (DIC) optics to count somite numbers during the somitogenesis period or trace the migration of lateral line primordia during the pharyngula period, which are key characters of staging in zebrafish embryos. In addition, the yolk mass becomes brittle when fixed, making it difficult to perform routine histological. For these reasons, 3‐D reconstruction from serial sections is not the optimal technique for studying bitterling development.

This study has shown that application of microCT is a highly efficient technique for studying rosy bitterling development. The volume rendering of X‐ray tomography is sufficient to virtually display the staging features of interest (compare for example the left and right columns in Figure 8). Virtual slices provide microanatomical tissue details (e.g. retinal lamination; Figure 24), and provide us with the ability to reconstruct complex 3‐D structures that were previously only visible through dye‐injection methods (e.g., the semicircular canal and alimentary tract in Figure 27).

## 5 CONCLUSIONS

This paper represents one of the first detailed studies of development in any teleost species using microCT. To define stages, we have used numeric characteristics such as somite number and prim‐number, which facilitate comparison with zebrafish stages, and more broadly facilitate the study of evolution and development in other teleosts.

## AUTHOR CONTRIBUTIONS

Wenjing Yi and Michael K. Richardson conceived the study. Wenjing Yi performed all embryological studies, including microCT analysis. Martin Rücklin helped with microCT studies. All authors helped Wenjing Yi with interpretation of the microCT data. Wenjing Yi prepared the manuscript and figures. Michael K. Richardson helped edit the manuscript.

## CONFLICT OF INTEREST

The authors declare that they have no competing interests.

### PEER REVIEW

The peer review history for this article is available at https://publons.com/publon/10.1002/jmor.21335.

## Supporting information


**Figure S1** *Rhodeus ocellatus*, development of the pharyngeal cartilages. (a and b) 235 hpf; (c and d) 330 hpf stage. Volume renderings (grayscale) with cartilage are segmented in a different color. (a and c) Ventral views, rostral to the left. (b and d) Lateral views, dorsal towards the top, rostral to the left. Abbreviations: cb, ceratobranchial cartilages; ch, ceratohyal cartilage; ep, ethmoid plate; hs, hyosymplectic; Mc, Meckel's cartilage; n, notochord.; pq, palatoquadrate; te, pharyngeal teeth. Scale bar, 200 μm

## Data Availability

All data are available on request from the first author.
