# Supplementary figures and images for: Normal stages of embryonic development of a brood parasite, the rosy bitterling Rhodeus ocellatus (Teleostei: Cypriniformes)
Source: J Morphol. 2021 Apr 2;282(6):783–819. doi: 10.1002/jmor.21335 (PMC8252481; doi:10.1002/jmor.21335)

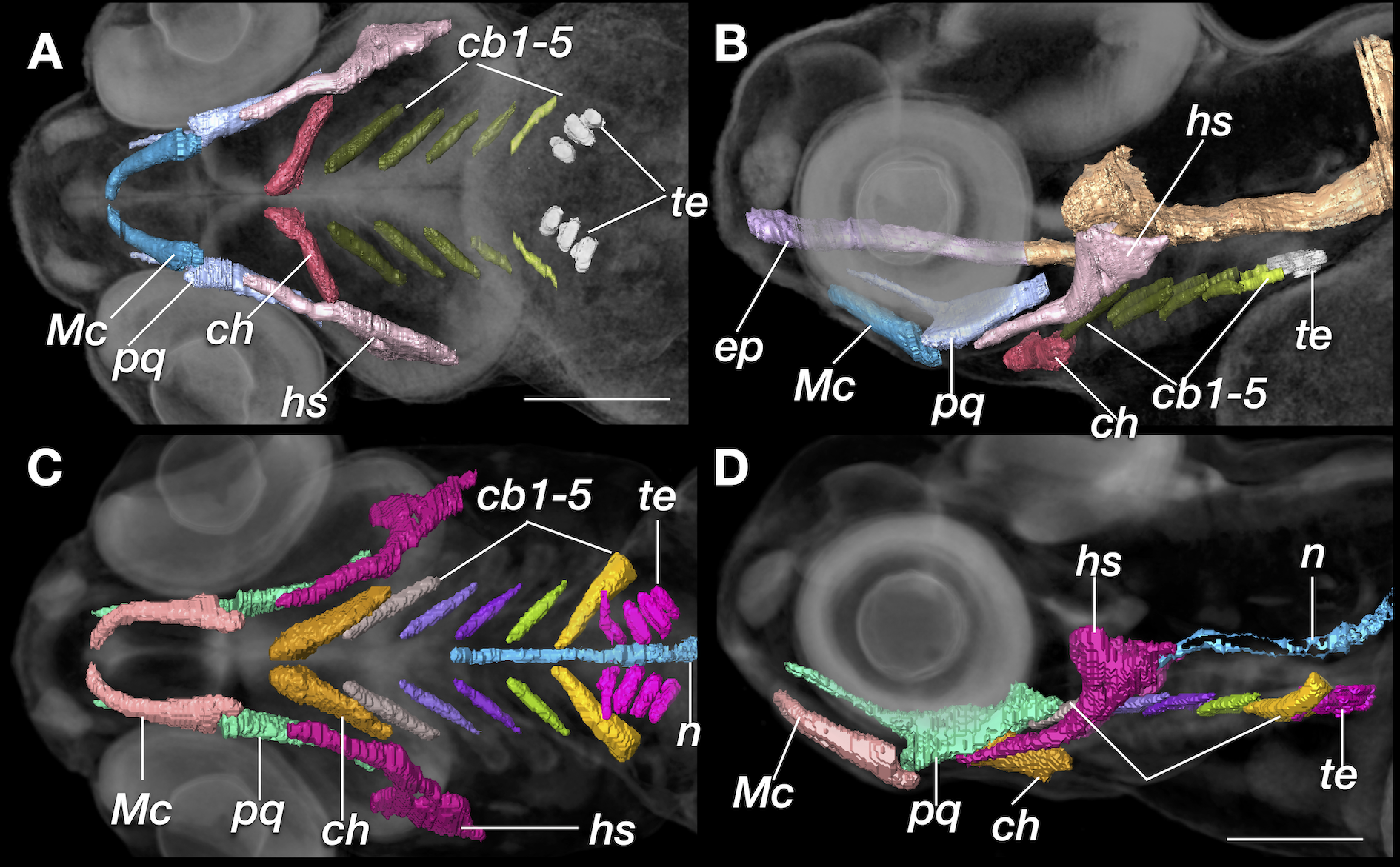

Supplement: Supplementary file 1 — Figure S1 Rhodeus ocellatus, development of the pharyngeal cartilages. (a and b) 235 hpf; (c and d) 330 hpf stage. Volume renderings (grayscale) with cartilage are segmented in a different color. (a and c) Ventral views, rostral to the left. (b and d) Lateral views, dorsal towards the top, rostral to the left. Abbreviations: cb, ceratobranchial cartilages; ch, ceratohyal cartilage; ep, ethmoid plate; hs, hyosymplectic; Mc, Meckel's cartilage; n, notochord.; pq, palatoquadrate; te, pharyngeal teeth. Scale bar, 200 μm [file JMOR-282-783-s001.tiff]
